# Exploring Generative Artificial Intelligence and Data Augmentation Techniques for Spectroscopy Analysis

**DOI:** 10.1021/acs.chemrev.4c00815

**Published:** 2025-06-23

**Authors:** Aaron R. Flanagan, Dhairya Dalal, Frank G. Glavin

**Affiliations:** † School of Computer Science, 8799 University of Galway, Galway City, Co. Galway H91 FYH2, Ireland; ‡ Data Science Institute, 8799 University of Galway, IDA Business Park, Galway City, Co. Galway H91 AEX4, Ireland

## Abstract

Generative artificial intelligence (AI) techniques are advancing rapidly and are becoming increasingly challenging to implement. Researchers, practitioners, and enthusiasts alike now require an understanding of complex concepts far beyond the scope of simple feed-forward neural networks to implement the current state-of-the-art methods for their research interests. In contrast, while data augmentation methods may not perform at the same level, they are easier to understand and implement, and are well demonstrated. For these reasons, this review aims to bridge the knowledge gap between the sciences of chemometrics and generative AI and provide a starting point for new researchers. In the context of spectroscopy, this work collects, categorizes, and describes the most popular preprocessing techniques and the state-of-the-art in generative AI and data augmentation, spanning over 104 peer-reviewed journals and proceedings across 32 publishers and organisations. We provide intuitive explanations of the methods, highlighting their strengths and weaknesses, and we include graphical and practical examples of their applications.

## Graphical abstract

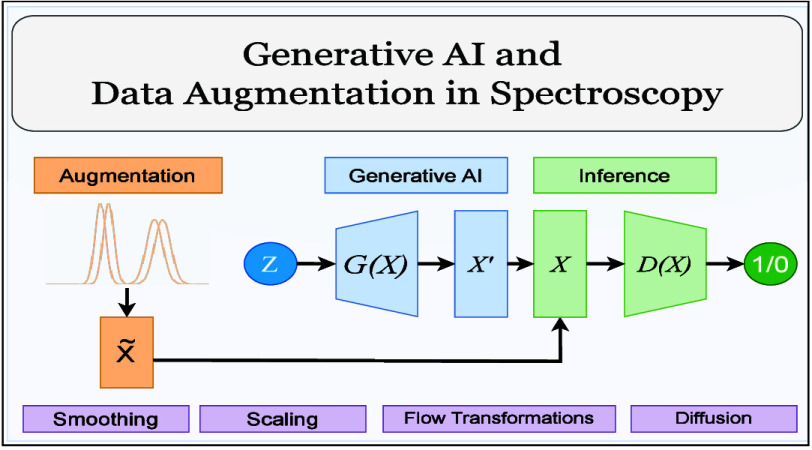

## 1 Introduction

Spectroscopy is the field of study concerned with the interactions between electromagnetic radiation and matter.^1^ These interactions can be categorized as the emission, absorption, reflectance, or scattering of light from a target sample at an atomic^2,3^ or molecular level.^4,5^ Complex spectra are recorded as a function of the frequency shift with respect to the incident radiation and analyzed to make observations about physical and chemical properties. The field of spectroscopy is broad, encompassing many techniques applied across a variety of problem domains, such as pharmaceutical development and analysis,^6,7^ gravitational wave monitoring,^8,9^ and food quality and safety,^10^ among many others.

Each spectroscopic technique encapsulates unique characteristics that affect the analysis, calibration, and prediction phases; however, certain limitations are present throughout them all. For example, curating large data sets is time-consuming and expensive, with a high probability that not all variations have been observed, and that noise is present.^11,12^ Complications can be introduced during calibration due to external factors such as cosmic rays, instrument noise, system geometry, or sample impurities obscuring the signal. As a result, calibration models tend to lack robustness because the sample set is limited and contains noise unrelated to the true chemical nature of the samples. Furthermore, for human-centric tasks that require personal health data, ethical and legal standards must be considered. These standards depend on the region where the data and practitioners operate and the regulatory bodies that govern the laws regarding fair data usage. Examples include the EU AI Act, General Data Protection Regulations (GDPR), and Goods Manufacturing Practices (GMP) for pharmaceuticals. These regulations are essential and must be upheld, but they impact the ability to collect, share, and analyze data in large quantities efficiently.

To overcome these limitations, data augmentation and generative artificial intelligence (AI) methods have been explored to expand the spectral database for training and calibration.^13–15^ Research in the domain has rapidly accelerated due to advances in generative AI such as Variational Autoencoders (VAE),^16^ Generative Adversarial Networks (GANs),^17^ and Diffusion models.^18^ Popular architectures such as BigGAN,^19^ StyleGAN,^20^ CycleGAN,^21^ and Denoising Diffusion Probabilistic Models (DDPM)^22^ have demonstrated excellent performance in generating high-fidelity and highly flexible models capable of producing realistic synthetic data. However, the cost associated with these advancements is a steep increase in the expertise and understanding required of the constituents that compose these large systems.

As with any problem, the challenge therein is the translation from the original domain implementation to the domain of interest. The complexity of these generative models has increased dramatically and now far surpasses the simplicity of standard feed-forward neural networks. For example, VAEs and GANs are designed to train two neural networks in tandem with the key differences relating to the latent space definitions, objective functions, and training procedures employed. The DDPM^22^ architecture, as the authors state, is inspired by nonequilibrium thermodynamics and requires a general understanding on the concepts of diffusion, Markov chains and variational (Bayesian) inference. The state-of-the-art has grown such that random experimentation to assess feasibility is not an easy task given that models now require extensive data and time to train. A practitioner must first have an understanding to implement such methods, and must also account for the cost and time, with considerations to their personal experience, available hardware, and proficiency in software.^23^

This review aims to bridge the knowledge gap between the sciences of chemometrics and generative AI. We provide a starting point for new researchers and a detailed explanation of the state-of-the-art in generative AI and augmentation techniques in the context of spectroscopy along with common preprocessing techniques typically applied beforehand. The contributions of this work include simplified mathematical descriptions, high-level pseudo code for algorithm implementation, intuitive visualizations demonstrating the effects of certain methods, and references to relevant studies demonstrating their application. Furthermore, we make available a complementary codebase for hands-on practical experience. The repository contains the data described below in section 2.3, along with code examples for performing preprocessing, augmentation, and training generative AI models. See the Data and Software Availability section for information about how to access it.

Lastly, this article assumes working knowledge of spectroscopy; we draw attention to alternative review articles with specific focus on spectroscopic techniques including *Raman* and *Surface-Enhanced Raman* (SERS),^24^ *Near-Infrared* (NIR),^25,26^ *Nuclear Magnetic Resonance* (NMR),^27^ *Proton Magnetic Resonance (MR)*,^28^ and *Laser-Induced Breakdown* *Spectroscopy* (LIBS).^29^ We also wish to highlight excellent review articles on machine learning and deep learning which contain detailed introductions on various algorithms and generative methods.^30,31^

## 2 Methodology

This section outlines the methodology employed to conduct and implement the review process. First, a brief cross-domain review was conducted to note existing augmentation and generative AI techniques to consider for inclusion. Some example preliminary results are presented in Table 1, and Figure 1 illustrates the coverage of the literature in this work according to type and publication year. Next, the research questions were defined to (i) narrate the scope of the article, (ii) outline the search criteria, and (iii) define the inclusion criteria. Lastly, a search strategy was designed which implements the use of customized query strings to retrieve the literature. The following sections will describe the research questions and search strategy in detail.

**Table 1:** Preliminary Results of Methods Listed to Consider for Inclusion

augmentation	generative AI
noising	diffusion
shifting	autoencoders
oversampling	normalizing flows
spectral blending	generative adversarial networks

**Figure 1:**
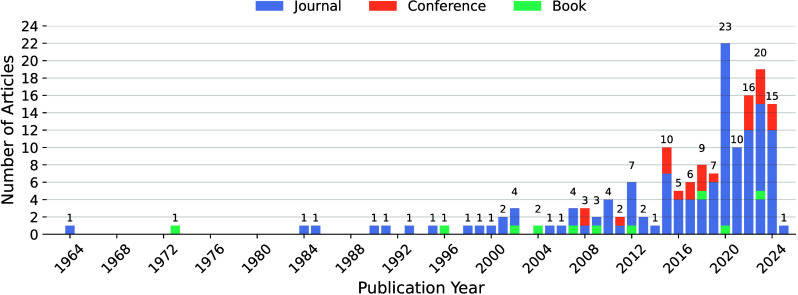
Distribution of covered articles by type (Journal, Conference Paper, and Book).

### 2.1 Research Questions

The goal of this study is to collect, categorize, and describe methods employed for the augmentation and generation of data in the domain of spectroscopy. This refers to any technique that manipulates existing data or produces new data for the purpose of training inference models. This includes data preprocessing, mathematical and statistical data augmentation, representation learning, and distribution approximations. The following research questions outline the focus of this study:

- Q1: What are the most popular preprocessing methods applied to spectroscopic data?
- Q2: What data augmentation methods have been proposed for spectroscopic data tasks?
- Q3: Which generative AI methodologies have been applied and evaluated for spectroscopic data?
- Q4: Which methods have demonstrated the most potential for further evaluation?

Research question Q1 will be discussed in section 3, Data Preprocessing. This section will expand on data pretreatments and subsequent preprocessing techniques typically conducted before augmentation and generative modeling. section 4, Chemometrics and Data Augmentation, will address Q2 and focus on topics categorized under conventional chemometrics and machine learning. Q3 is expanded on in section 5, Generative Deep Learning, which describes the current state-of-the-art in the generative AI domain. Finally, Q4 will be addressed in section 6, Conclusion.

### 2.2 Search Strategy

The goal of the search strategy is to extend the broad cross-domain review by incorporating relevant terms into the query strings. The search strings are constructed with Boolean operators and single-quotes to emphasize specific phrases and topics frequent in the literature, as presented in Figure 2. The primary databases accessed in this study to catalogue methods for inclusion are *IEEE Xplore*, *SpringerLink*, https://ieeexplore.ieee.org/Xplore/home.jsp, https://link.springer.com; *ScienceDirect*, https://www.sciencedirect.com; *PubMed*, https://pubmed.ncbi.nlm.nih.gov; *Semantic Scholar*, https://www.semanticscholar.org; and *Google Scholar*, https://scholar.google.com/. Methods identified during the preliminary search that have not been applied in the domain of spectroscopy were filtered out. The articles selected are based on the following inclusion criteria: (i) published in English, (ii) published after 2010, (iii) available full text, and (iv) peer-reviewed. Regarding point (ii), we endeavored to select up-to-date literature, with exceptions made for earlier works that remain relevant, especially where methods were first proposed. With regard to point (iii), this includes literature accessible through open-access or journals via institutional licenses.

**Figure 2:**
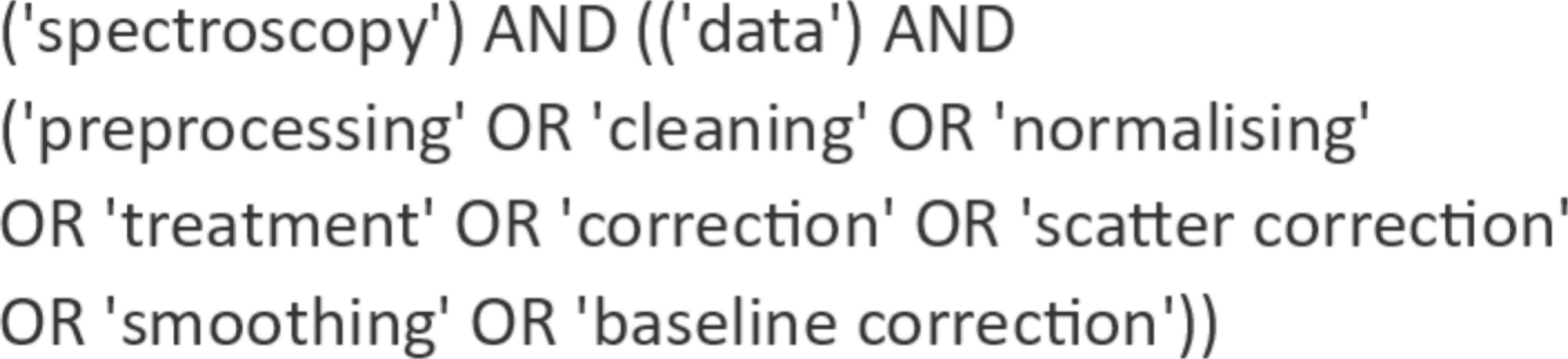
Query string applied for section 3, Data Preprocessing.

### 2.3 Graphics

We present graphical illustrations throughout this paper to demonstrate the techniques when applied to the Raman spectrum of pure *cyclohexane*. The data was collected using an *Endress+Hauser* Raman Rxn2 analyzer with a noncontact Rxn-10 probe.^32^ Cyclohexane was selected because of its popularity as a reference standard for calibrating equipment, such as a Raman spectrometer. Each signal spans wavelengths of 150–3245 cm^–1^ with 1 cm^–1^ resolution. For reference, the chemical structure of cyclohexane is C_6_H_12_, and the Chemical Abstracts Service (CAS) number is 110-82-7. Please refer to the Data and Software Availability section below for more details.

Figure 3 illustrates the best sample representation that was produced, while Figure 4 presents a small batch of 50 samples. The data were collected using the Raman Rxn2 analyzer and a single 4 mL glass vial with dimensions of 0.6 in. outer diameter and 1.8 in. height. The vial was fixed in place in a static analysis chamber and a 10× objective lens noncontact probe was moved laterally, and around its cylindrical axis, to capture the output signal of the sample chemical at various optical path lengths, including the probe being too close and far away. The plots and associated algorithms are implemented in Python v3.11 using the Matplotlib,^33^ SciPy,^34^ and NumPy libraries.^35^

**Figure 3:**
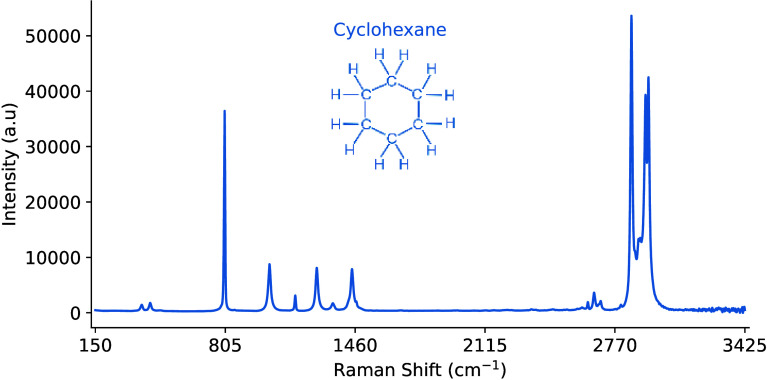
Example of the perfect Raman signal and structural formula for pure cyclohexane.

**Figure 4:**
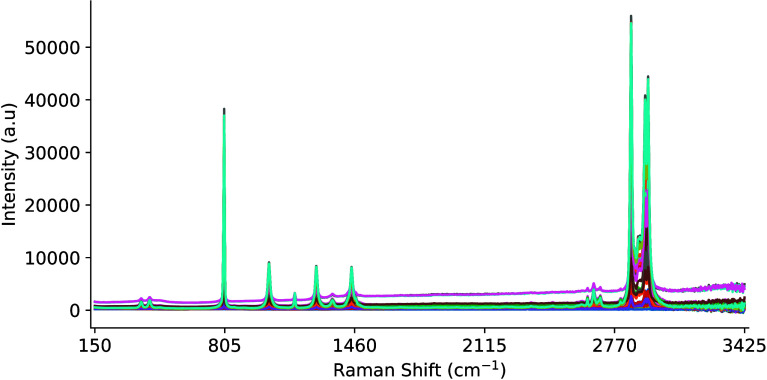
Batch of 50 Raman spectra of cyclohexane at various optical path lengths.

## 3 Data Preprocessing

Data preprocessing is the first challenge postdata collection and should be investigated before synthetic data generation. The purpose of preprocessing is to remove any external effects not related to the true chemical and physical nature of the sample.^36–38^ The aim of preprocessing is to remove scattering effects and baseline offsets introduced by (i) samples scanned by different analytical systems, (ii) changes in particle sizes, (iii) changes in path lengths, (iv) fluorescence, or (v) cosmic radiation, among others.^39,40^

The steps for executing a chemometric analysis project from experimental design to analysis have been described in excellent detail.^41^ Our work aims to be complementary, drawing attention to their implications while discussing the preprocessing methods that should be investigated prior to performing data augmentation and generative AI. Spectral data analysis and modeling can be divided into a three-step pipeline that defines the order of operations to clean the data and obtain the most useful information. These operations include the spectral pretreatment of the data after acquisition, optional but advised preprocessing techniques, and, finally, the data analysis and modeling.^42,43^

This section focuses on the first two pipelines: pretreatments and preprocessing. First, we briefly expand on two important topics: the implications of preprocessing with regard to augmentation and generative modeling and the data pretreatments. Then, we will expand on the second pipeline that incorporates popular preprocessing methods. This includes data scaling techniques, which are fundamental to many chemometric preprocessing algorithms and highlight their relation to similar concepts in deep learning, and important preprocessing techniques such as scatter correction and detrending.

### 3.1 Implications

It is important to consider the implications that preprocessing has on the distribution of the data. The order of the processing methods affects the quality of the preprocessed data. Each preprocessing technique alters the data in a unique way and presents its own benefits and drawbacks. For example, data scaling is usually applied to minimize outliers and increase computational efficiency by shrinking the data between lower numerical bounds. However, if not applied correctly, it may amplify false representations such as noise or distort the underlying distribution, as demonstrated below in Figure 6. The preprocessing method selected should give respect to the original spectroscopic data. Furthermore, most machine learning workflows assume the data are independent and identically distributed (iid), such that a random variable is not influenced by others, or through memory, and is sampled from the same underlying probability distribution. To this end, preprocessing should be applied consistently to all the data; that is, the training and testing sets undergo the same preprocessing steps. After such, it is important to validate that the preprocessing method did not introduce any new artifacts that alter the underlying assumptions and biases and that all preprocessed data still adhere to the assumption of iid. Data augmentation and generative AI applications should not be applied before identifying such risks. This stems from a common saying in data science: “*Garbage In, Garbage out*”. The output of such models is directly linked to the quality and representation of the input data.

### 3.2 Spectral Pretreatments

#### 3.2.1 Cosmic Ray Removal

Cosmic rays are random high-intensity particles that collide with a detector during spectral acquisition and introduce sharp peaks into the spectral data. These are often magnitudes larger than the intensity of the pure signal and can obscure the true measurements. Cosmic ray removal, or despiking, refers to the operation of identifying and removing the nonsample related artifacts from the signal.^42^ This operation should be performed as a first step prior to other pretreatment and preprocessing methods. Removing the spikes prevents them from affecting subsequent processing steps. Some options include spike removal algorithms that operate independently over the spectrum such as median filtering,^44^ wavelet transforms,^45^ and polynomial fitting,^46^ or multispectrum algorithms that require a spectral data set such as deep learning CNN models, 2D Laplacian filtering, outlier detection in intensity distributions, or special software such as Renishaw’s WiRE for example.^47^ Many analytical instruments will automatically perform cosmic ray removal, along with methods such as dark current subtraction, which is the case with the Raman Rxn2 analyzer applied to collect the data for this work.

#### 3.2.2 Wavenumber Calibration

Spectrometers are regularly calibrated to ensure that the measured spectra remain accurate and consistent over time. Wavenumber calibration is a key step in this process, and it is applied to account for a phenomena referred to as peak shifting. Peak shifting, also known as peak drift, horizontal shift, frequency shift, or spectral shift, refers to the event where peaks in a spectrum appear at unexpected wavelengths. This shift can occur toward either lower wavelengths (blue-shift) or higher wavelengths (red-shift). Many factors may result in peak shifts, examples include (i) instrumental and environmental variations such as temperature change, random instrumental noise, or calibration drift, or (ii) sample variations due to changes in analyte concentration, pH levels, physicochemical interactions, electronegativity, path length, or bond lengths.^48–50^ The goal of wavenumber calibration is to correct the peak shifts along the *x*-axis of the signal and to perform an accurate wavenumber assignment. This enables the comparison of data across various data sets and analytical systems and helps improve qualitative and quantitative performance. This may be performed in the instrument during acquisition, typically along with intensity calibration, or it can be applied after acquisition via shift correction and calibration algorithms.^51,52^

#### 3.2.3 Baseline Correction

Baselines represent the background of a spectrum in the absence of informative features, such as peaks, acting as reference levels in silent regions of the signal. The background signal should ideally be a flat line on or near zero, but it may be distorted due to variations such as instrument drift, scattering effects, detector noise, or sample imperfections. Baseline correction is a fundamental pretreatment that should be performed following data acquisition and before chemometric analysis. It represents the goal of the preprocessing methods discussed in this work, which aim to remove nonsample-related artifacts that obscure the true signal. This step helps eliminate the unwanted variations in the spectrum, ensuring that it is clean and interpretable, thus improving quantitative and qualitative analysis. Many correction techniques are available, each depending on the type of spectroscopic data and the complexity of the baseline. For example, Raman spectra are characterized by sharp peaks, and baseline correction is often used to remove fluorescence effects. In contrast, NIR spectra typically exhibit broad features, and baseline correction is applied to remove low-frequency drift with sharp variations most likely attributed to noise. Choosing the correct approach to baseline correction is important, some techniques include asymmetric least-squares, robust baseline estimation, wavelet transforms, and polynomial fitting.^53^ Furthermore, the Savitzky–Golay filter described in this work, along with scatter correction techniques such as the multiplicative scatter correction (see Figure 10) and standard normal variate, described below, can be applied to correct baseline drifts.^36^

#### 3.2.4 Cropping

Cropping, also referred to as spectral truncation,^41^ is typically applied before downstream preprocessing methods like normalization, described below in section 3.3, to preserve data integrity and reduce the risk of distorting the signal with irrelevant information. The goal is to retain the informative wavelength regions while discarding irrelevant areas, such as those lacking absorption features or noise introduced by the detector during acquisition.^54,55^ Removing these regions reduces noise and improves calibration performance, as chemometric and machine learning models perform better when trained on the main spectral features. Cropping can be done manually, requiring domain expertise to select relevant spectral ranges, or automatically, using statistical techniques or machine learning to identify key regions learned through inference. However, it is important to consider when cropping is appropriate. For example, in Raman spectroscopy, higher wavenumber regions (e.g., > 1800 cm^–1^) may still contain valuable information, particularly for organic compounds.

### 3.3 Data Scaling

Normalization and standardization are simple techniques that are applied in most preprocessing applications such as scatter correction, derivative preprocessing, and detrending. Data scaling is considered a de facto standard when training deep learning models to overcome the computationally heavy and vast amounts of time and resources that are required. Typically, the input data are scaled so that the original observations fit some desired range or assumed distribution, e.g., a standard Gaussian with mean zero and unit variance. This is referred to as feature scaling and serves the purpose of reducing the computational overhead when calculating large quantities of weighted sums, nonlinear functions, and error gradients during training. It also includes other benefits such as applying regularization during training to prevent issues with exploding or vanishing gradients.^56^ This is when the gradients become too large and result in very unstable weight updates or when the gradients become so small that the initial layers in the model do not update and it is unable to converge to an optimal solution. Furthermore, feature scaling enables a comparison of data from various analytical systems and can help improve the robustness of calibration models.

#### 3.3.1 Min–Max Normalization

Min–max normalization scales the values in a sample between a specified range per feature, i.e., the column of intensity readings at a specific wavelength, assuming the data is ordered tabular.^57^ This transformation is low-cost and applied to all the columns independently to ensure that no particular feature range dominates others and that each feature contributes equally. The main benefits presented through this method include the preservation of relative distance and reduction of variance locally in each feature, and the reduced computational overhead during model training. In terms of memory and computational requirements, it is more efficient to compute the nonlinear activations and gradients of deep learning models using scaled numerical inputs. A major drawback of this technique is that min–max does not manage outliers efficiently and can often magnify them.

To scale data via min–max, first calculate a divisor range by subtracting the maximum and minimum occurring values over the column, i.e., across all the samples at that specific wavelength. Each intensity value is then iteratively subtracted by the minimum and divided by the range. This scales the feature values between 0 and 1, when applying the standard formula, as shown in eq 1:

$$X_{\mathrm{scaled}}=\frac{X-X_{\min}}{X_{\max}-X_{\min}}$$ 1

where *X* refers to the full column of feature values, *X*_min_ is the single smallest occurring value, and *X*_max_ is the single maximum intensity observed. It is also possible to select an arbitrary user-defined range for the lower and upper limits, as demonstrated in Figure 5.

**Figure 5:**
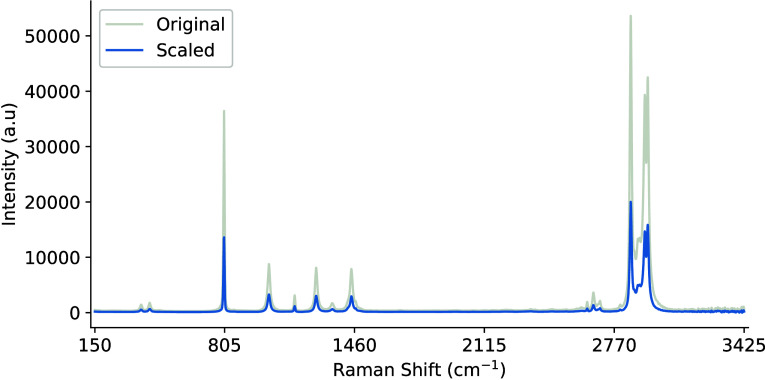
Min–max normalization scales the values in the signal to range between a user defined threshold [0–20,000].

A less common variation of this method is max normalization, which is applied per sample, i.e., per row, by dividing each value in the signal by the maximum intensity observed. This method roughly scales the values between 0 and 1 (eq 2):

$$X_{\mathrm{scaled}}=\frac{X}{X_{\max}}$$ 2

A major benefit with this approach is that it operates locally per sample and does not incorporate information from the distribution. This limits the influence that outliers have on the scaling effect. However, the caveat is that it does not consider the relative distance and variance of the features, and as a result, the distribution of each feature is not preserved.

#### 3.3.2 Standardization

An alternative to scaling the features is to transform the data to fit a Gaussian distribution. Standardization, also referred to as *z*-score normalization, transforms the original data distribution so that the observations are centered around the mean with unit variance *N*(0,1). Standardizing a data distribution is a simple process which involves subtracting the distribution mean from each sample and using the standard deviation as the scaling factor (eq 3):

$$z=\frac{x-\mu }{\sigma }$$ 3

where *x* is the sample score, μ is the distribution mean, and σ is the standard deviation.

In contrast to min–max normalization, the relative distance and variance of the features are not preserved, which can result in distortions in the signal. Outliers may also affect the transformation process and introduce a skew in the transformed distribution. It is not advised to standardize the entire distribution when dealing with spectral data, as demonstrated in Figure 6. This method is applied more appropriately per sample, which is further elaborated upon in section 3.4.1, Standard Normal Variate.

**Figure 6:**
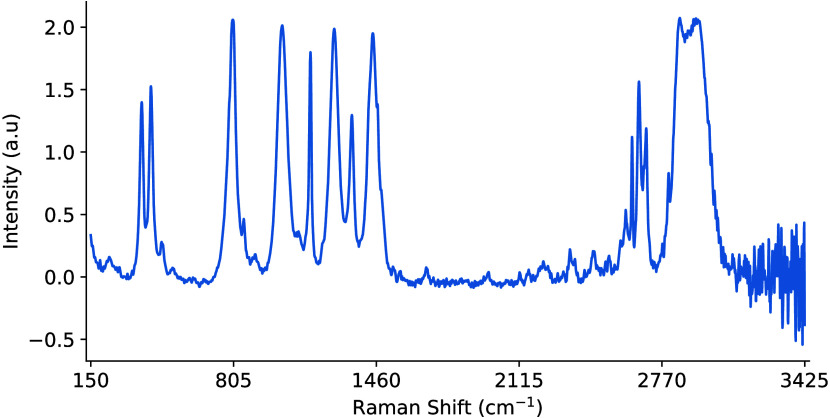
Effect of standardization when applied over a distribution of 145 samples of cyclohexane. This includes perfect examples, some with sloping baselines, shifts, and other noisy variations. The data distribution is now centered with μ = 0 and σ = 1. Peak location, shape, and magnitude are not maintained as a result.

#### 3.3.3 Neural Regularization Strategies

Neural regularization strategies refer to a growing body of regularization methods that have been introduced as layer constructs in neural network-type architectures. Normalizing data before training aids in reducing training costs, but issues such as vanishing gradients, as described in section 3.3, Data Scaling, still occur due to the large number of calculations on gradients conducted in the process. Vanishing gradients have a significant impact on performance, so to circumvent these issues, scaling techniques are often incorporated into model architectures to scale and maintain the data distribution as it flows and transforms through the network.

Batch Normalization (BatchNorm)^58^ is a regularization technique applied to increase stability and improve convergence speed when training neural networks. BatchNorm transforms the inputs of each layer by recentring and rescaling them using the current mini-batches mean and variance. When consistently applied throughout the network, this reduces the internal covariate shift, an issue in neural networks where the distribution of inputs to a layer changes during training. By reducing the internal covariate shift and ensuring the inputs to each layer follow a consistent distribution, the learning process is then simplified, which results in faster and more stable training.

Prior to input for any hidden layer, BatchNorm first computes the mean (eq 4) and variance (eq 5) for each feature *i* in *x* independently over instances in a mini-batch *B* of size *m* that is processed through the network, where *B* = [*x*_1_, ..., *x*_*m*_]:

$$\mu_{B}=\frac{1}{m}\overset{m}{\underset{i=1}{\sum }}x_{i}$$ 4

$$\sigma_{B}^{2}=\frac{1}{m}\overset{m}{\underset{i=1}{\sum }}{\left(x_{i}-\mu_{B}\right)}^{2}$$ 5

The output values of the features are then centered and scaled by subtracting their respective batch mean and dividing by the standard deviation (eq 6):

$${\mathrm{x̂}}_{i}=\frac{x_{i}-\mu_{B}}{\sqrt{\sigma_{B}^{2}+\epsilon }}$$ 6

where ϵ is a constant added to the variance for numerical stability. Two more parameters are introduced to increase the representation power of the model (eq 7): gamma (γ) and beta (β). Both parameters are optimized during training and function as scaling and shifting factors, respectively.

$$y_{i}=\gamma {\mathrm{x̂}}_{i}+\beta$$ 7

These parameters provide the network with the ability to center the feature on a learned mean and scale the variance. Lastly, the application of BatchNorm is slightly different when running inference. During inference, the output should depend only on the input and not on any particular batch. To overcome this, a moving average is calculated over all of the batch mean and variances and applied.

Layer normalization (LayerNorm)^59^ was proposed to replace BatchNorm in recurrent models with varying sequence lengths. BatchNorms performance is contingent on the size of the mini-batch, which is not fixed or well-defined for sequences of varying length. Instead, LayerNorm is applied per sample, making it batch invariant, and it is calculated across all features in layer *l*. The key distinction is related to averaging over the number of hidden units *H*, as opposed to batch size, for the mean and standard deviation:

$$\mu^{l}=\frac{1}{H}\overset{H}{\underset{i=1}{\sum }}x_{i}^{l}$$ 8

$$\sigma^{l}=\sqrt{\frac{1}{H}\overset{H}{\underset{i=1}{\sum }}{\left(x_{i}^{l}-\mu_{l}\right)}^{2}}$$ 9

Each activation output in the layer is normalized in a similar fashion to BatchNorm, by subtracting the layer mean (eq 8) and dividing by the standard deviation (eq 9) with a small epsilon value added for stability, as previously shown in eq 6. LayerNorm also has the benefit that its application in training and testing remains constant.

Two alternative techniques that are proposed to correct covariate shift while remaining batch invariant are instance normalization (InstanceNorm)^60^ and group normalization (GroupNorm).^61^ These methods apply the same normalization steps per sample per feature and per sample over a group of features, respectively. It should be noted that for 1D spectral signals, the data are typically ordered in a 2D tabular format, where each spectrum represents a row and the feature, i.e., wavelength, is the column. The application of each method is presented in Figure 7 to provide some intuition. The true strength of the methods can be observed when applied to imaging data or in convolutional neural networks (CNN) where the methods operate over the channel dimensions of the learned feature maps.

**Figure 7:**
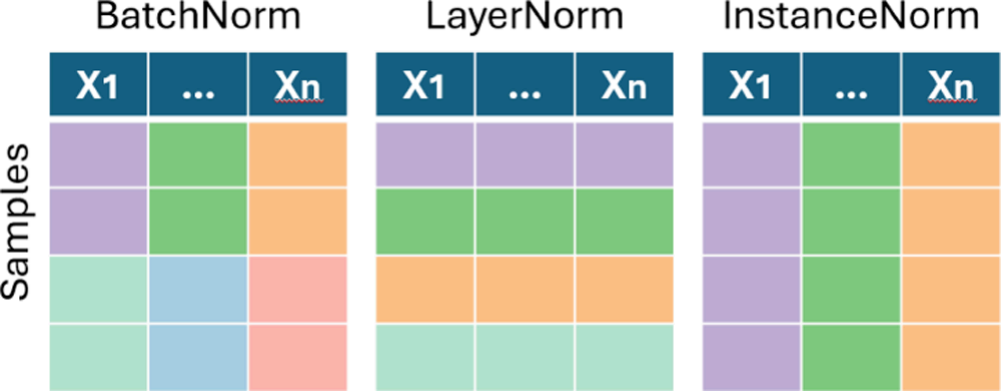
Application of normalization methods on 2D tabular ordered spectra. Each color represents an independent operation over the features and samples.

A benefit of deep learning and the previously described methods is the ability to learn complex nonlinear patterns, ignore noise, and process the data in a format optimized for the task at hand. Learning the optimized normalization routine through training and inference is possible, and less manually intensive, than standard preprocessing. This connection will be more apparent when we describe how chemometric preprocessing algorithms, described below, translate to equivalent operations in deep learning models. However, for difficult tasks, preprocessing the data and applying regularization strategies may be required to achieve robust models.

### 3.4 Scatter Correction

Scattering effects account for a large portion of noise in recorded spectra.^36^ These effects can be categorized as additive and multiplicative. Additive effects present as a baseline offset, shifting the baseline vertically along the *y*-axis, while multiplicative effects displace and scale the overall shape and peaks in the signal, as demonstrated in Figure 8. Variations such as temperature shifts, laser intensity, optical path variations, sample size, particle size, physical conditions, and sample positioning can influence the true signal.^40,62,63^ The goal of scatter correction methods is to apply some statistical assumptions regarding the nature of the sample and find the best algorithm to approximate such variations and remove them, which may prove difficult for some tasks.^64^ Scatter correction methods may lead to the loss of fine-scale information, as demonstrated below in section 3.5.1. This is especially important in regression settings, where the concentration of the analyte directly corresponds to the intensity of the signal.

**Figure 8:**
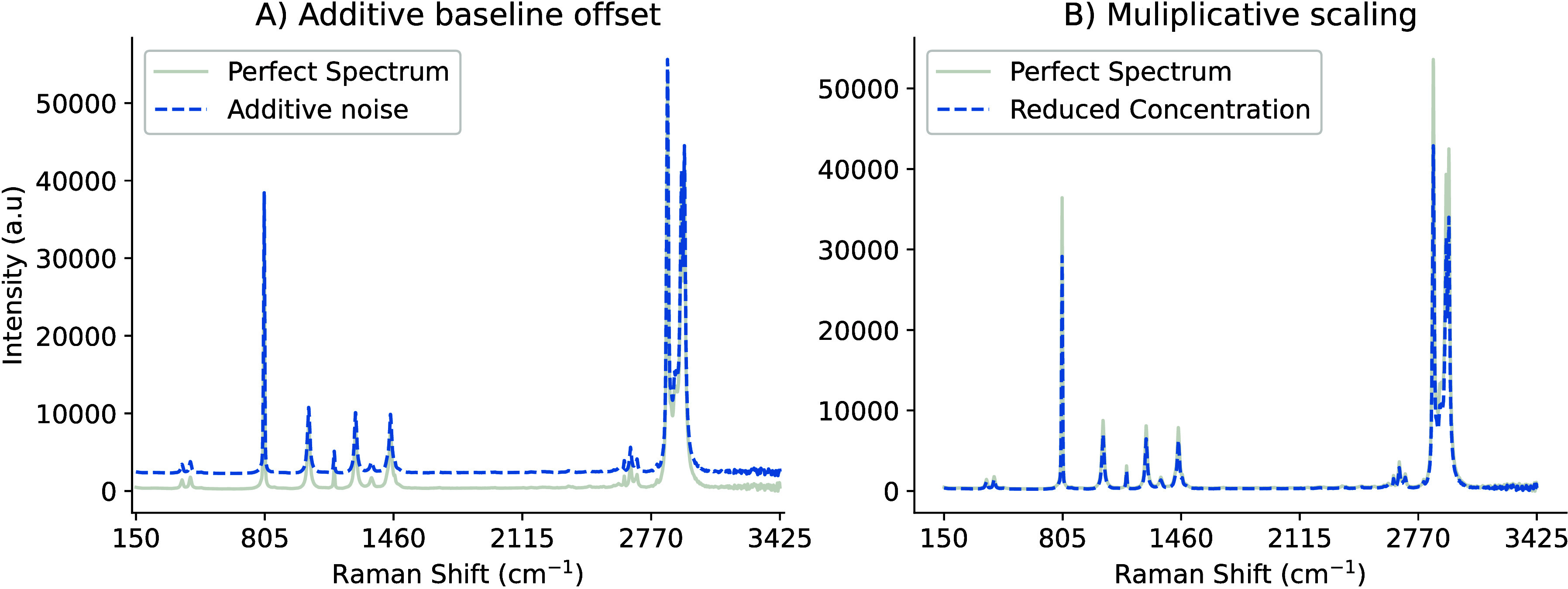
(A) Example of additive noise, via increased intensity by 2000 au, such as instrumentation variation introducing a constant offset across the baseline, and (B) multiplicative spectral scaling effect resulting from reduced concentration (80%).

#### 3.4.1 Standard Normal Variate

Standard normal variate (SNV) is a frequently applied chemometric preprocessing technique to reduce the baseline shift, varying global intensities, and the influence of multiplicative scattering effects in the spectral signal.^65–68^ SNV applies row-wise standardization, transforming each sample to have a mean of 0 and a standard deviation of 1. Each spectrum is normalized (eq 3) by subtracting its own mean (eq 8) and dividing by its deviation (eq 9), where *H* in this context represents the number of wavelengths for some random sample *x*. As a result, the normalized samples will be directly comparable in terms of intensity, as demonstrated in Figure 9, along with a reduction in the random variation present.

**Figure 9:**
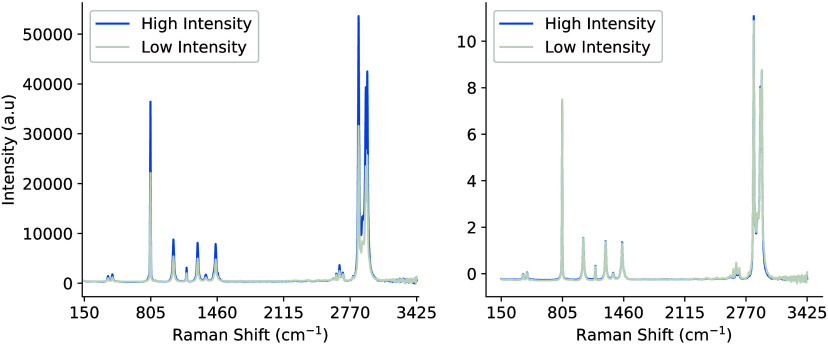
Example spectra before and after SNV normalization: The left side presents two example Raman spectra of cyclohexane with high (Figure 3) and relatively lower intensity. The right side demonstrates the comparability between the spectra after normalization.

The final point we wish to highlight is the similarity between the SNV algorithm and the LayerNorm implementation in fully connected neural networks (FCNN). In preprocessing, SNV is applied directly to the data prior to calibration using per-sample statistics. In deep learning, LayerNorm performs the same normalization procedures on the nonlinear weighted sums of the signal, i.e., using the layer activation outputs and statistics. These operations are equivalent when LayerNorm is applied directly after the input layer and before the first hidden layer in an FCNN. Each neuron represents an input variable, so the layer statistics will be equivalent to the sample statistics. LayerNorm also has the added benefit that it incorporates learnable parameters that can adjust the mean and variance during training. However, this similarity is valid only at the input layer and only for FCNN models. The LayerNorm implementation is typically computed over the channel’s axis when applied to higher-dimensional feature maps in deep architectures such as the CNN.

#### 3.4.2 Multiplicative Scatter Correction

Multiplicative scatter correction (MSC),^69^ also referred to as multiplicative signal correction, is an algorithm proposed and widely employed^63,67,68,70^ for reducing additive and multiplicative scattering effects^37^ such as path length differences (see Figure 10). It stems from the Lambert–Beer law that describes the absorptivity and resulting spectral signal of a sample relative to its optical path length.^71^ MSC first requires that an estimate of the ideal spectrum free of scattering effects is provided as a parameter, or under the assumption that the shape of each spectrum is similar, the average spectrum can be computed. This technique corrects spectra by computing a linear least-squares (eq 10) over each sample to the mean spectrum. First, each sample *i* in *X* is fit to the mean spectrum X̅ over *K* wavelengths (eq 10):

$$X_{i}=a_{i}+\overset{K}{\underset{k=1}{\sum }}b_{ik}\cdot {\mathrm{X̅}}_{k}+e_{i}$$ 10

where *a*_*i*_ and *b*_*i*_ represent the additive baseline offset (intercept) and weights (multiplicative scaling factors) coefficients, respectively, and *e*_*i*_ is the residual error between the actual and fitted signal. Then, a least-squares regression is applied to estimate the coefficients *a*_*i*_ and *b*_*i*_ for each sample *X*_*i*_ by minimizing the residual sum of squares. Once the coefficients are estimated, each sample *X*_*i*_ is replaced with its corrected signal using the coefficients (eq 11):

$$X_{i}=\frac{X_{i}-a_{i}}{b_{i}}$$ 11

**Figure 10:**
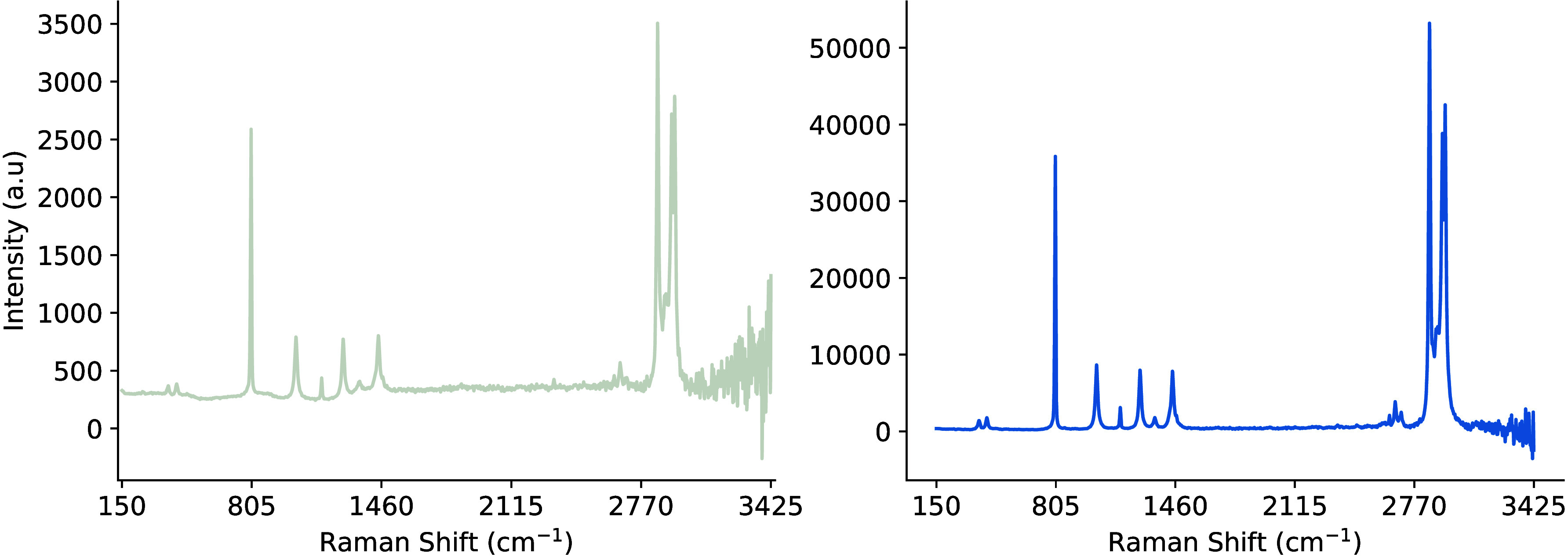
MSC demonstrated the ability to successfully correct low intensity samples with sloping baselines and scattering effects when a perfect representation (Figure 3) is applied as reference.

In summary, the goal of the MSC technique is to preserve the true spectral features while simultaneously removing noise and distortions from the signal. Mathematically, it shares similarities with neural networks as both involve computing a weighted sum of inputs and a bias (intercept) to learn weights or coefficients that approximate the data. Furthermore, they both aim to simplify downstream tasks, such as calibration. Either approach is capable of performing preprocessing and requires consideration of which approach is optimal. MSC is deterministic, making it reproducible, less-costly to compute, and based on well-defined principles. One potential drawback is that MSC may remove variability that could be useful for calibration. As a result, consistent preprocessing is required when new data are introduced into downstream tasks. In contrast, deep learning models are more flexible. They can learn the variability in the data, allowing them to automatically filter out noise in new data when they are sent downstream. However, achieving this data-specific correctional behavior requires significant effort. During initial training, deep learning models are nondeterministic, meaning their outcomes will vary. Developing a model that balances training performance with generalization to new data is both time-consuming and computationally expensive. A large and diverse data set that contains this variability is required to achieve robust generalization. In the absence of diverse data, CNN architectures such as Residual Networks (ResNet)^72,73^ can be utilized to leverage existing information via residual connections to improve gradient flow and training efficiency. While this is not a substitute for diverse data sets, it can help simplify training and enhance performance.

##### 3.4.2.1 Extended Multiplicative Signal Correction

The authors of MSC proposed an enhancement entitled extended multiplicative signal correction (EMSC) to improve path length estimation by including prior knowledge of the spectra.^71,74,75^ This technique (eq 12) builds upon MSC by incorporating polynomial coefficients to be estimated in the least-squares regression. The motivation behind this enhancement states that baseline offsets are often additive, e.g., considering fluorescence in Raman spectra, so the resulting spectrum may contain a sloping baseline. Incorporating and estimating polynomial coefficients allows the method to correct this phenomenon.

In EMSC, a matrix representing the polynomial trends *P* up to degree *d* is defined as part of the reference model. This matrix includes the reference spectrum X̅ and the subspace of all spanning column vectors representing the spectral wavelengths *k* for each polynomial term *p* applied, where [ X̅ , *p*_0_, *p*_1_, *p*_2_, ···, *p*_*d*_]. Fitting a single sample *X*_*i*_ to the reference spectra involves the same approach as MSC, projecting the spectrum onto the subspace, but now includes the polynomial trends (eq 12):

$$X_{i}=a_{i}+b_{i}\cdot \mathrm{X̅}+d_{1}p+d_{2}p^{2}+\cdot \cdot \cdot +d_{n}p^{n}+e_{i}$$ 12

When the polynomial trend is zero, the spanning vector represents a constant offset of 1 which is scaled by the estimated coefficient *d*_0_. With the inclusion of *d*_0_ from the polynomial trends matrix, the additive offset *a* can be removed from the equation, and it can now be defined as (eq 13):

$$X_{i}=b_{i}\cdot \mathrm{X̅}+\overset{d}{\underset{j=0}{\sum }}d_{j}p^{j}+e_{i}$$ 13

where *b*_*i*_ represents the multiplicative coefficients, X̅ is the reference spectrum, *e*_*i*_ is the residual term, *d*_*j*_ are the polynomial coefficients, and *p* ^*j*^ represents the polynomial trend vectors. The spectra are then corrected (eq 14) similarly to MSC, now including the polynomial coefficients:

$$X_{i}=\frac{X_{i}-\overset{d}{\underset{j=0}{\sum }}d_{j}p^{j}}{b_{i}}=\mathrm{X̅}+\frac{e}{b}$$ 14

To provide an example of the matrix, if a quadratic term is selected, the trends to be corrected in the signal include a zeroth-degree polynomial *P*_0_(*k*) = 1, which is a constant offset, a first-degree linear trend *P*_1_(*k*) = *k* and the quadratic *P*_2_(*k*) = *k* ^2^. The resulting matrix would be defined as illustrated in Figure 11.

**Figure 11:**
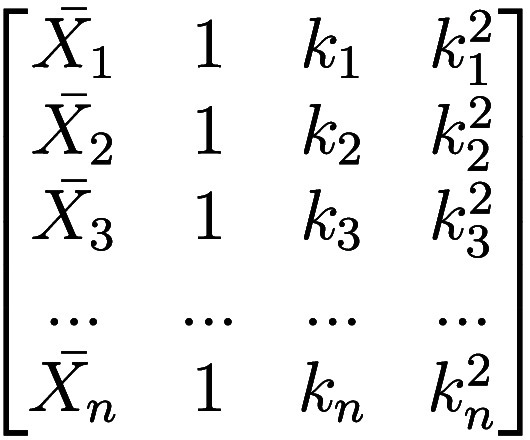
EMSC matrix formulation of polynomial terms.

#### 3.4.3 Orthogonal Signal Correction

In the context of orthogonal signal correction (OSC),^76^ the algorithm steps are quite large and require understanding of the Partial Least Squares (PLS)^77^ and Nonlinear Iterative Partial Least Squares (NIPALS)^78^ algorithms. In this review, we opt not to expand on the method in detail due to the required prerequisite knowledge of the topics, however, due to the number of applications and frequent references,^79–81^ we believe it should be highlighted for new researchers.

OSC is a preprocessing technique proposed to extend on PLS inspired methods to remove components in a spectral set *X* that are orthogonal and unrelated to a set of measured responses *y*. Briefly, PLS is an algorithm that reduces the number of predictor variables in *X* while maximizing the explained covariance in *y*. It achieves this by calculating latent variables from weighted linear combinations of the predictors. An orthogonalization step is then performed to ensure they remain uncorrelated, and the variables are sorted in descending order of explained covariance. OSC differs from the PLS approach by calculating latent variable weights that minimize the covariance and approaches orthogonality, thus highlighting and removing the unrelated variation in *X* with regard to *y*. It applies this in an iterative manner inspired by NIPALS to remove one component from *X* at a time.

### 3.5 Detrending

Detrending and scatter correction are distinct concepts for preprocessing data prior to calibration. However, in the literature, authors may refer to methods such as normalizing and SNV as detrending, where others may refer to them as separate methods that can be paired, causing some confusion. Detrending as a concept is more clearly defined when discussing time series analysis with a focus on removing subtrends from the data. In these cases, two distinct approaches are discussed: (i) detrend by difference and (ii) detrend by model fitting. Subtrends in 1D spectral analysis typically refer to baseline offsets and slopes.^57^ As a technique, detrending can also refer to the standalone method for fitting a polynomial to the signal and subtracting the coefficients to reduce baseline shift and curvilinearity in the sample signal.^82^ In these cases, it is similar to the SNV and MSC algorithms, such that software package functions (e.g., in *R* or *Python*) and articles may automatically assume and incorporate a normalization step as part of the process. Typically, the aim of detrend functions is to remove linear subtrends, which as mentioned, translate to baseline offsets in 1D spectral signals.

#### 3.5.1 Smoothing

Smoothing, also referred to as low-pass filtering, is the process of attenuating high-frequency noise fluctuations present in the signal.^57^ The simplest technique that can be applied is moving average smoothing (MAS), which reduces noise by substituting the measured intensities at each wavelength λ_*i*_ with an average calculated over a fixed window *w* of size 2*w* + 1. The window convolves over the sample one wavelength at a time until the full length of the signal *k* is traversed^83^ (eq 15):

$$\lambda_{i}=\frac{1}{w}\overset{k}{\underset{i=-k}{\sum }}\lambda_{i+k}$$ 15

Other smoothing approaches include weighted average, Gaussian, median, and exponential smoothing, along with polynomial and derivative methods discussed below. It is important to note that smoothing algorithms may contain side-effects such as the loss of important and fine-scale information,^84^ as illustrated in Figure 12.

**Figure 12:**
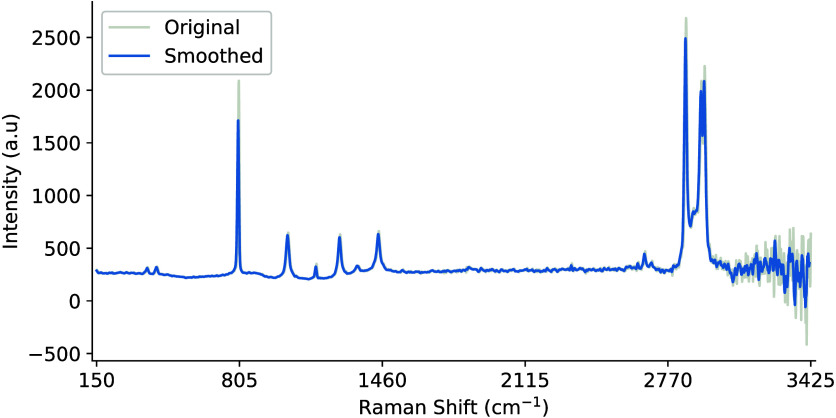
Moving average smoothing with window size 7 applied to reduce scattering effects between wavenumbers of 2900–3425 cm^–1^. Increasing window length results in smoother outputs, but also effects the magnitudes of the peaks, removing fine-scale information.

#### 3.5.2 Derivatives

Derivative preprocessing methods are examples of high-pass filters that amplify high-frequency features such as peaks obscured by baseline variation and noise.^36^ The most popular and widely adopted algorithm is the Savitzky–Golay (SG) filter.^85^ The SG filter can be described as an enhancement of the MAS and detrend algorithms that further incorporates derivatives. The user determines the window size and order of the polynomial and derivative to calculate in advance. Its primary application is to smooth data by calculating the coefficients of a polynomial on successive subsets of the data points. When a derivative is not selected, the SG filter essentially acts as a low-pass smoothing filter, and the central point of the window is then replaced with the fitted value from the estimated coefficients optimized via least-squares. However, it can also be applied as a high-pass or band-pass filter^86^ by calculating the derivative of the central point using the polynomial coefficients, allowing it to attenuate low-frequency signals and amplify the high-frequency components, as illustrated in Figure 13.

**Figure 13:**
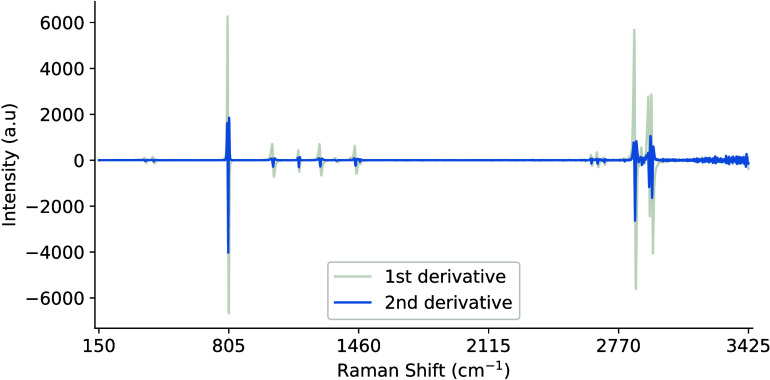
Example first and second derivative signals generated via SG filters with a second-order polynomial and window length of 5.

Norris–Williams (NW) derivation^87^ is an alternative derivative preprocessing technique. NW adopts the concept of finite differences to enhance the spectral resolution and reduce the level of noise inflation in the signal. This involves two steps: (i) smooth the spectra over a window of points (eq 15), and (ii) for first-order derivation, calculate the difference between two smoothed values with a selected gap size [*i* – gap, *i* + gap], or for second order derivations, take double the smoothed value *i*, add the smoothed value *i* + gap, and then take the difference with the smoothed value *i* – gap.^36^

#### 3.5.3 Transforms

Transforms are powerful techniques that have revolutionized signal processing due to their ability to convert complex signals between the temporal, frequency, and spatial domains, with the latter being applied in the case of imaging techniques. The most popular method among these has also been incorporated into the apparatus for collecting spectral data,^88–90^ the Fourier transform (FT). The idea behind the FT is to represent any complex signal as a sum of smaller frequency components. It converts a signal from its original domain, typically time or spatial, into the frequency domain by decomposing the signal into a sum of sinusoids (sine and cosine functions).^39^ Each component represents a unique frequency, and their amplitude characterizes their presence in the signal. This enables practitioners to identify and remove undesired noise in the signal by applying filters to attenuate either high or low frequencies. The smoothed signal can then be reconstructed from the denoised signal by applying the inverse Fourier transform (IFT), as shown in Figure 14.

**Figure 14:**
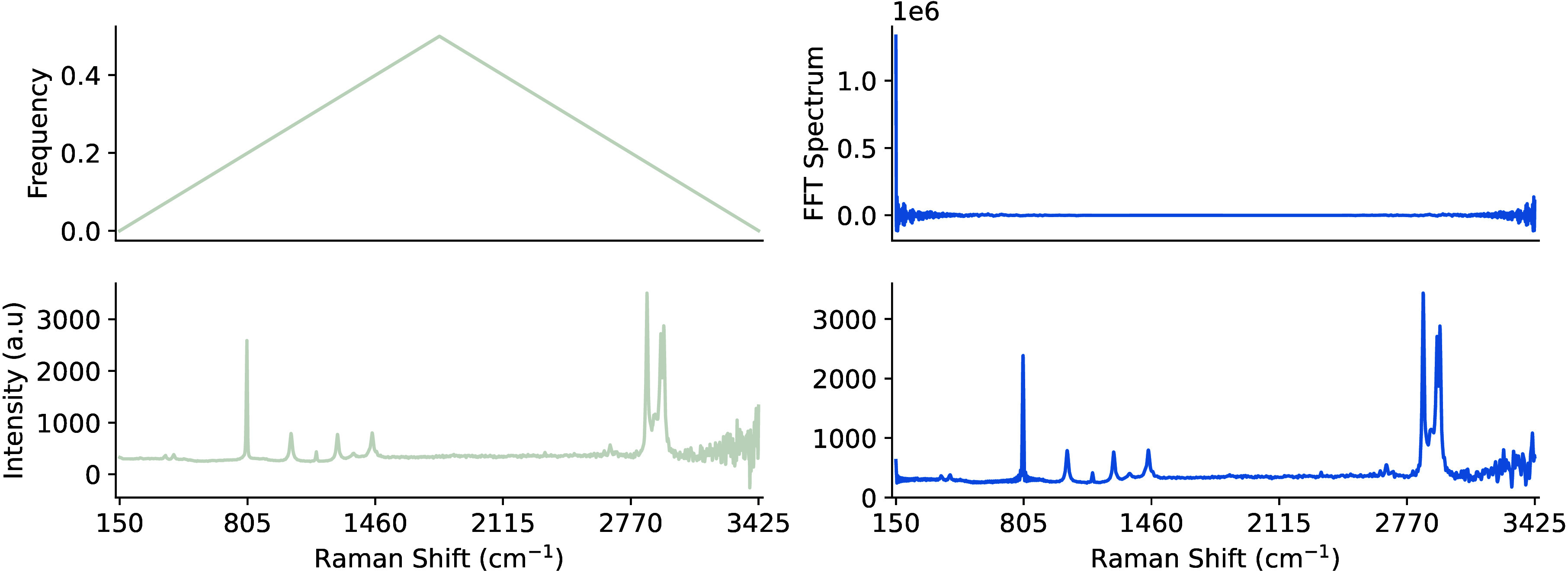
Fourier smoothed Raman spectra showing the absolute values of the frequency bin centers (top-left), the FT spectrum (top-right), original sample (bottom-left), and the smoothed spectrum (bottom-right) when a simple low-frequency threshold filter (<0.1) is applied.

Wavelet transforms (WT) are a family of algorithms designed to extend and improve on FT filters.^91^ WT filters have the ability to represent the signal in the temporal or spatial domains, along with the frequency domain, and perform multiresolution analysis of a signal. Each WT technique has a defined basic wavelet termed the mother wavelet, and the basis function wavelets are derived through the scaling and translation of this prototype. Like FT filters, applications of the WT filters can be employed as band-pass filters for baseline correction, noise reduction, and feature enhancement. Wavelets at various scales and translations are applied to extract frequency-like information from a signal, where low-frequency (baseline) and high-frequency (noise) can be identified and attenuated to clean the signal. The processed signal can then be reconstructed using the inverted wavelet transform (IWT). WT techniques can be categorized under two approaches: (i) continuous wavelet transform (CWT), and (ii) discrete wavelet transform (DWT), a sampled version of CWT that utilizes discrete sets of scales and translations. Furthermore, many families of WTs exist that apply different mother wavelet functions as their basis, such as Haar, Hilbert, Daubechies, Symmelet, and more.^39^

## 4 Chemometrics and Data Augmentation

The terms synthetic data and data augmentation are often used interchangeably; however, they refer to different concepts with the same goal. Data augmentation refers to a body of techniques that modify existing data to increase diversity and variation in the original set. In contrast, the term “synthetic data” refers to any new data that are artificially generated to simulate real-world examples. Regardless of the terminology, the goal is to produce new data that model unseen variations and improve the performance of calibration models.

Machine learning and deep learning algorithms have been adopted under the umbrella of chemometric analysis and are considered the state-of-the-art in the field, with deep learning pioneering the state-of-the-art in performance. The most effective methods are generally supervised learners that require substantially large data sets for training to ensure inference with a high level of confidence. However, data scarcity is often a recurring challenge due to the cost of time and resources required for producing large sets of high-quality data. To address this issue, literature has recently focused on deep generative modeling architectures for synthetic data generation. While state-of-the-art, generative models are technically complex and often suffer from unstable training, making such methods challenging for general adoption. Alternatively, nondeep learning-based algorithms for data augmentation are still relevant and can provide benefits which include increased interpretability, lower computational resource requirements, and smaller experimental run-times. This section expands on various machine learning and statistical algorithms employed to perform data augmentation for spectral data.

### 4.1 Over- and Undersampling

Imbalanced data is a common challenge for machine learning algorithms, where the training distribution is highly skewed because the target categories are not equally distributed. Training on imbalanced data can result in unintentional model bias toward the more frequent phenomena, resulting in diminished classification prowess for minority categories. To address this issue, techniques such as oversampling and undersampling can be employed to correct the class distribution and account for the imbalance. Oversampling is primarily used to increase the representation of the minority class in imbalanced data sets, but it can also be applied to any class that requires additional representation. Similarly, undersampling is most commonly applied to decrease the representation of the majority-class but follows the same principle. It can be applied to other classes where a decrease in representation is required. These techniques can shift the class prior probabilities toward a uniform distribution, aiming for balanced representation during training. The sampling process involves randomly selecting data from the original set until a user-defined number of samples or class balance ratio is achieved. This can be achieved through sampling without replacement, where the data are removed from the set after sampling, or with replacement, where the data are not removed and can be selected again.

The Synthetic Minority Over-sampling Technique (SMOTE) is an algorithm that generates synthetic data for the minority class using information from the feature space.^92^ For each sample in the minority class, SMOTE employs the k-Nearest Neighbors (kNN) algorithm to interpolate between the feature vectors of the instance and its *k* nearest neighbors, operating over the line-segments that connect them in their feature space.^93^ The number of neighbors and synthetic samples produced are determined based on the desired oversampling rate. New synthetic samples are produced by calculating the difference between the feature vector of the instance and randomly selected neighbors, scaling these difference vectors with a random value between 0 and 1, and adding the scaled difference vectors to the instance feature vector. After oversampling of the minority class is performed, undersampling of the majority class through random removal is repeated until a predefined class balance ratio is achieved. A limitation of SMOTE is that it assumes that interpolation between samples is semantically meaningful and that minority class instances are clustered in the feature space. Additionally, the interpolation is also linear, which does not scale well with conventional machine learning methods, and if the minority class is densely clustered, generating synthetic data will increase this density, not offering much variation.

Adaptive synthetic sampling (ADASYN) is an extension of SMOTE proposed for generating synthetic data to reduce the bias presented by class imbalance and to adaptively estimate and shift the classifier discrimination boundaries toward the difficult to learn minority samples.^94^ ADASYN calculates the class imbalance and the number of synthetic samples required to achieve a user-defined balance ratio. Then for each sample, it calculates the density distribution of the *k* nearest neighbors to identify which minority samples are most surrounded with the majority, i.e., those near the decision boundary and difficult to learn. The majority–minority ratio corresponds to the difficulty weight assigned per sample, which will be adaptively updated. The number of synthetic samples generated for each minority class sample is proportional to its difficulty weight and the required number of synthetic samples in total, which is determined via the user-defined balance ratio. This ensures that more synthetic samples are generated for the minority class.^95–97^ Other proposed extensions of SMOTE applied in spectroscopy include borderline SMOTE,^98^ Tree-SMOTE-XGBoost,^99^ GKIM,^100^ and TwoWin-SOVA.^101^

### 4.2 Noise

Inference models are trained primarily on representative data, and as a consequence they may not generalize to new samples that deviate from the true nature of the problem. Adding noise to the original data serves the purpose of augmenting the data to increase the diversity of the training set, while also applying regularization to improve the robustness of the model to slight jitters and variations that could naturally occur. Noise can be modeled to simulate many expected phenomena, such as background noise, scattering and instrumental effects, and statistical perturbations.^102^ The most common application of noise is sampled from the probability density function of a normal distribution (eq 16):

$$f\left(x\right)=\frac{1}{\sigma \sqrt{2\pi }}\;e^{-\frac{1}{2}{\left(\frac{x-\mu }{\sigma }\right)}^{2}}$$ 16

where μ and σ represent the distributions mean and standard deviation. The noise is sampled with the same dimensionality as the spectra and added to each wavelength element-wise.

Furthermore, noise can be applied to simulate different spectral acquisition scenarios that may contain additive, multiplicative, sloping, instrument, or intensity effects.^11,103,104^ It is also important to note that noise may have a negative impact on the inference model if it is not applied appropriately. The noise added between wavelengths in the spectrum is typically considered statistically independent because it is based on the observed standard deviation. However, sample and system variations can result in different deviations around the mean across each wavelength.^105^ In these circumstances, investigation and alternative means for modeling the problem should be considered.

### 4.3 Peak Shifting

Peak shifting, as described above in section 3.2.2, refers to the event where wavenumber calibration is required to perform wavenumber assignment and correct for the drifts of the peaks in the signal. In the context of data augmentation, peak shifting modifies the data to simulate real-world variations, as demonstrated in Figure 15. By exposing a model to slightly shifted versions of the spectral data, it helps the model become more robust to natural shifts in peak positions. However, this should not be considered a substitute for wavelength calibration and shift correction. Many proposed shift correction methods respect the original data assumptions and should be considered before applying it as an augmentation strategy.

**Figure 15:**
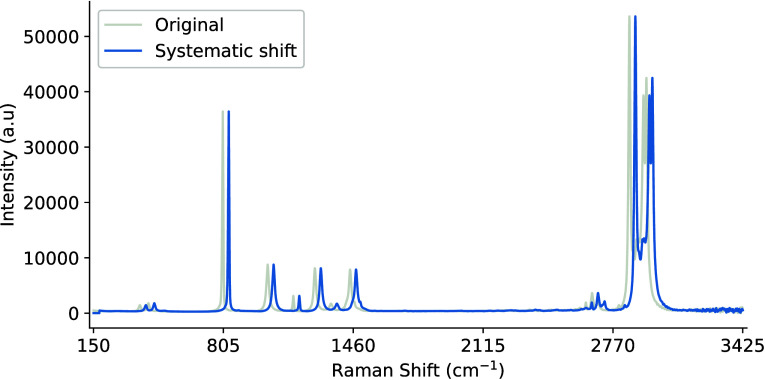
Example uniform peak shift on a Raman spectra sample of pure cyclohexane by 50 reciprocal centimeters (cm^–1^).

### 4.4 Extended Multiplicative Signal Augmentation

Extended multiplicative signal augmentation (EMSA) is a technique to generate synthetic data by introducing physical variations modeled on observed data using the EMSC algorithm (see section 3.4.2.1).^14^ The EMSC method aims to reduce scattering effects by estimating the coefficients for additive *a*, multiplicative *b*, polynomial *d*, and residual terms *e*, and apply them for spectral correction. To generate data via EMSA, EMSC is first performed on each spectrum to the reference sample to estimate the parameters and their standard deviations (σ_*a*_, σ_*b*_, and σ_*d*_). Gaussian noise, termed the deviations (Δ*a*, Δ*b*, Δ*d*), is then sampled per parameter by conditioning a Gaussian distribution on the standard deviation of the parameter with μ = 0. Lastly, for each observed spectrum that was fit, we add the noise to the parameters, e.g., *a*′ = *a* + Δ*a*, and compute the new augmented spectrum (eq 17) by updating eq 12 to include the new parameters:

$$X^{\prime }=a^{\prime }+b^{\prime }\cdot \mathrm{X̅}+d_{1}^{\prime }p+d_{2}^{\prime }p^{2}+\cdot \cdot \cdot +d_{n}^{\prime }p^{n}+\frac{e\cdot b^{\prime }}{b}$$ 17

where *X*′ is the new augmented sample, X̅ is the reference spectrum, and *b*′ and [*d*_0_′, *d*_1_′, ···, *d*_*n*_′] are the new parameters with added deviations.

### 4.5 Intensifying

Spectral intensifying^12^ was proposed to reduce baseline variations introduced by scattering effects when spectra are collected from different instruments. This approach scales the wavelength values in a signal *A* by an amplification factor *M* and a baseline factor *C*. Intuitively, when *M* = 1 the values will not be scaled, so setting *C* to any value except *C* ≠ 0 allows the user to simulate a baseline offset at various extremes. For the inverse scenario, setting *C* = 0 and *M* ≠ 0 is equivalent to a multiplicative baseline offset.

$${\mathrm{Sample}}_{\mathrm{new}}={\left\{A_{\mathrm{new},k}\right\}}_{\left(k=1..n\right)}=\left(M*A_{\mathrm{original},k}\right)+C$$ 18

The intensifying process (eq 18) involves multiplying each wavelength value *k* in *A* by an arbitrarily selected multiplicative factor *M*, then adding the baseline offset constant *C*. The algorithmic steps (Algorithm 1) to apply the intensifier are provided in Chart 1.

**Chart 1:**
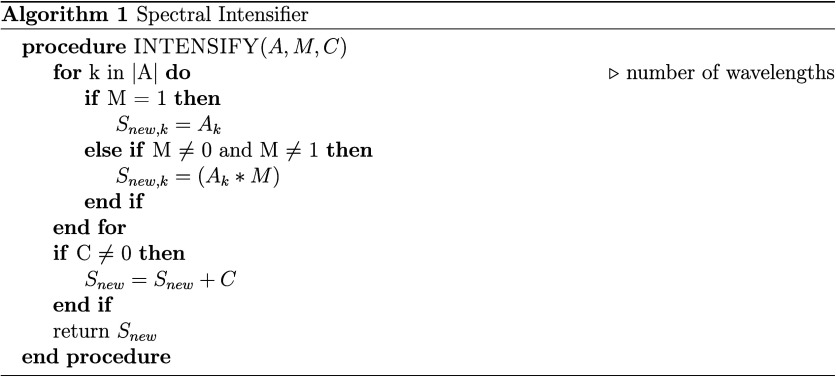


### 4.6 Blending

Spectral blending^12^ generates synthetic data by computing weighted averages over unique batches of samples to simulate admixtures with varying concentrations. To perform spectral blending (eq 19), first a set of weights referred to as the concentration grades are defined that will be used to scale each sample. For each sample *A* in a batch *m* calculate the product of the value at wavelength *k* and the respective samples concentration weight per_i_, for *k* = 1 to *n* wavelengths, and sum the results over *m* samples. The average is then calculated by dividing the weighted sum of *m* by the sum of the concentration weights ∑_*i* = 1_^*m*^ per_*i*_.

$${\mathrm{Admixture}}_{\mathrm{new}}={\left\{A_{\mathrm{new},k}\right\}}_{\left(k=1..n\right)}=\frac{\overset{m}{\underset{i=1}{\sum }}{\mathrm{per}}_{i}{*A}_{i,k}}{\overset{m}{\underset{i=1}{\sum }}{\mathrm{per}}_{i}}$$ 19

where *A*_new,*k*_ is the weighted averages for each specific wavelength *k* over *m* samples.

Blended synthetic data were further expressed as fractional proportions of unique pairs of samples where the concentration grades sum to one, removing the necessity to calculate an average over each batch. In this scenario, the synthetic spectra modeled a linear interpolation between samples across the distribution.^106,107^ The amount of synthetic data generated increases exponentially in correlation to the number of weights and original samples available (eq 20):

$$S_{\left[1,...,|W|\right]}=\forall w_{i}\in W\;(w_{i}*A)+\left((1-w_{i})*B\right)$$ 20

where each weight *w*_*i*_ in *W* is multiplied by all the observed values in *A*, and the weight 1 – *w*_*i*_ is multiplied by the observed values in *B*, and the two results are summed. This produces five new spectral signals, when for example *W* = {0.1, 0.3, 0.5, 0.7, 0.9}, for each unique pair, and |*W*|*N*(*N* – 1)/2 total synthetic samples, where *N* is the number of original samples. This method is applied individually for each class of samples in the training data. We simplify this by expanding and presenting the algorithmic steps to produce new spectra when blending two spectra *A* and *B* in Algorithm 2 (Chart 2).

**Chart 2:**
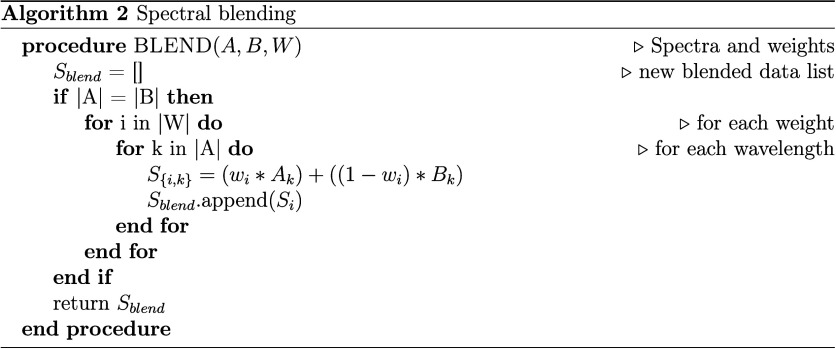


Spectral blending is efficient at generating large amounts of data. However, if the original data set contains class imbalances, this augmentation method will further exacerbate the imbalance due to the exponential increase in synthetic data as a product of the blending procedure. For example, lets define classes A and B to have 100 and 50 samples, respectively, and define 9 weights ranging between 0.1 and 0.9. Applying the formula ((|*W*|* *N*) * (*N* – 1))/2 independently for both classes to calculate the number of synthetic samples generated, indicates the number of new samples per class is 44550 and 11025, with a total of 55575. After concatenation of the synthetic data with the original data, the class balance ratio, which was initially 66–33%, is now approximately 80–20%. In such scenarios, stratified sampling to maintain the original class balance, or under-sampling to realize an equal balance, is recommended.

### 4.7 Hybridization

Hybridization is an augmentation method similar to spectral blending for generating synthetic data using nonrepeated random information mixing of unique pairs of real-world data.^108^ Given a set of real-world spectral data *X*, and their corresponding target outcomes *Y*, each new synthetic sample is generated by taking the average over the sum of two spectra (eq 21):

$$X_{\mathrm{SSD}}=\frac{1}{2}\left(X_{i}+X_{j}\right)$$

$$Y_{\mathrm{SSD}}=\frac{1}{2}\left(Y_{i}+Y_{j}\right)$$ 21

where *X*_*i*_ and *X*_*j*_ are the random spectral samples and *X*_SSD_ is the hybrid sample representing the average of the two parents. The authors hypothesize that this method can produce spectral gradients in proportion to natural occurrences, in this context leaf nutrients estimated via inductively coupled plasma-optical emission spectroscopy (ICP-OES), and expand the knowledge base for training inference models. The high-level algorithmic steps to perform hybridization are presented in Algorithm 3 (Chart 3).

**Chart 3:**
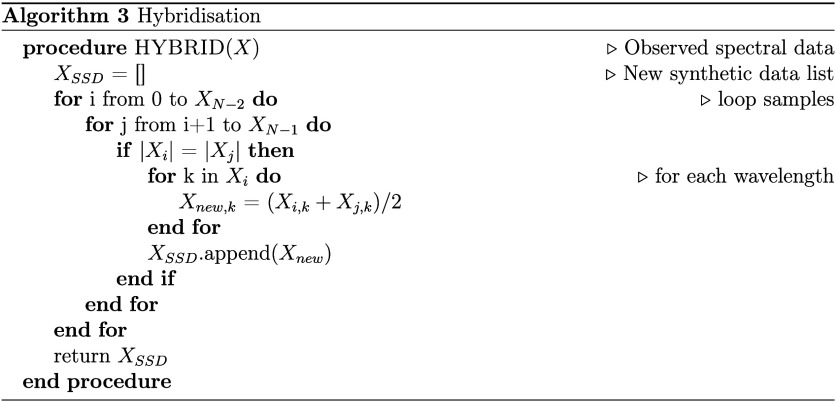


### 4.8 SDA-Fusion

Spectral data augmentation fusion (SDA-Fusion) is designed to combine the synthetic outputs of many data augmentation algorithms.^109^ The framework ensembles multiple augmentation techniques to generate synthetic data under variable conditions and assumptions, resulting in more diverse data than data generated from any particular method alone. The augmentation techniques employed in this framework include Gaussian noise addition, random variation from slope, multiplication and offset, SMOTE, random wavenumber shifting, an FCNN GAN, and a convolutional GAN. The fusion spectral data set is produced through concatenation of all the synthetic data from each method.

## 5 Generative Deep Learning

Generative deep learning models for synthetic data generation are becoming more powerful. While these advances enable researchers to produce higher quality data, this increase in performance has an associated cost with regard to the knowledge, resources, and time required for implementation, and also warrant certain considerations.^110^ Another important point to consider is that synthetic data is artificially generated based on a subset of data for any specific task, and these samples are not guaranteed to represent the true data distribution. Therefore, it is the practitioners’ duty to ensure the synthetic data is representative, realistic, and reproducible through natural causes and observations. This section presents the most popular generative deep learning methods used for synthetic data generation in spectroscopy and provides intuitive explanations regarding their underlying foundations.

### 5.1 Autoencoders

An autoencoder (AE) is an encoder–decoder neural network architecture that was initially designed to perform feature extraction and data reconstruction. The encoder learns to map input data to a lower-dimensional representation referred to as the latent space, and the decoder learns to sample from this space and reconstruct the input.^111^ Many extensions have been proposed in a variety of domains to enhance the AE in feature learning, dimensionality reduction, and data reconstruction,^112–114^ which can be utilized to produce synthetic data. For vanilla AE models, the encoder–decoder functions are constructed as neural networks parametrized by independent weights *w* and a bias *b*. The encoder function *g*(.) translates input representations through hidden layers mapped to output latent variable vectors (eq 22):

$$z_{i}=g\left(w_{e},b_{e};x_{i}\right)$$ 22

where *x*_*i*_ is the input sample, and *z*_*i*_ is the latent vector representation. The decoder function *f*(.) takes *z*_*i*_ as input and up-samples to reconstruct the original input (eq 23):

$${\mathrm{x̂}}_{i}=f\left(w_{d},b_{d};z_{i}\right)=f\left(g\left(x_{i}\right)\right)$$ 23

where *x̂*_*i*_ represents the reconstructed sample. The model is trained to minimize a reconstruction loss function *J*, such as the mean squared error (MSE) (eq 24):

$$J_{\theta }=\frac{1}{n}\overset{n}{\underset{i=1}{\sum }}{\left(x_{i}-{\mathrm{x̂}}_{i}\right)}^{2}$$ 24

where for each sample *n*, the average squared difference between the reconstructed and original sample are calculated. Squaring the result removes negative values and also provides the benefit of penalizing larger errors. The function parameters θ refer to the weights and biases in the encoder and decoder functions, and by minimizing the loss function, they are optimized.

A limitation in vanilla AEs is that the encoder network maps inputs to fixed points in the latent space, which results in the model becoming deterministic. Furthermore, spectral data sets are often complex and intractable to approximate, resulting in synthetic outputs with limited variation. Variational Autoencoders (VAEs) overcome this by incorporating variational inference, a Bayesian method for approximating intractable distributions through optimization (see Figure 16). In the context of VAEs, given a model with parameters θ, and some observed data *X*, the true posterior distribution *p*(θ|*X*) is typically unknown and intractable to compute. Instead, a simple and tractable prior distribution, such as a standard Gaussian, for example, is defined over latent variables *p*(*z*). The aim is to approximate the true posterior *p*(*z*|*x*) with a variational posterior *q*(*z*|*x*), which is learned via the encoder network. The variational posterior represents the updated beliefs regarding the latent variables after observing the real data. This process involves maximizing the evidence lower bound (ELBO), which represents a lower-bound on the log-likelihood of the observed data given that the current set of distribution parameters in the variational posterior is the best fit. By maximizing the ELBO, the variational posterior *q*(*z*|*x*) becomes a learned distribution shifted as close to the true posterior *p*(*z*|*x*) as possible, while simultaneously maximizing the log-likelihood.

**Figure 16:**
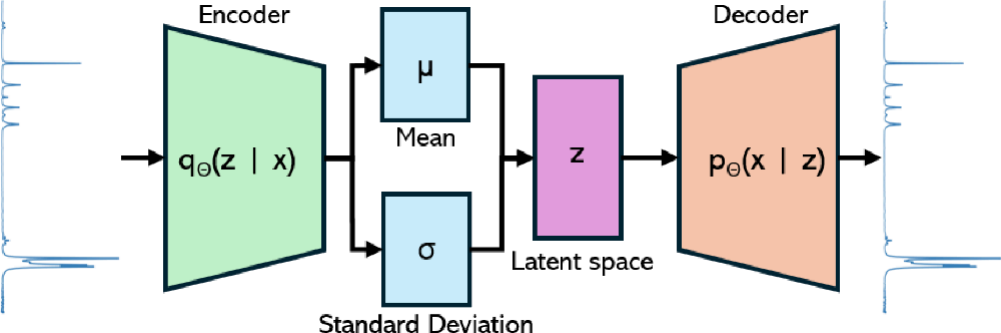
High-level example of VAE architecture.

In practice, the latent space is constructed using two more layers that can be optimized, and they represent two sets of parameters, the mean μ and the standard deviation σ. These parameters will define the distribution from which the latent variables are sampled. Now, instead of learning a fixed mapping from *z* to *x*, the latent space is modeled as a variational posterior distribution (eq 25):

$$q_{\theta }\left(z|x\right)=N\left(z;\mu ,\sigma \right)$$ 25

where *q*_θ_(*z*|*x*) represents the conditional probability distribution of the latent variable *z* given *x*, parametrized by θ. The encoder with parameters θ models the conditional distribution as a Gaussian *N*(*z*; μ,σ) and learns to output parameters μ­(*x*) and σ­(*x*). The latent vector *z* is then sampled from this distribution using the learned parameters. However, sampling from a distribution is nondifferentiable, so in order to enable backpropagation the reparameterization trick is applied. The reparameterization trick models the sampling process as a deterministic and differentiable function, allowing for gradients to be calculated during training. It also includes element-wise multiplication with independent Gaussian noise ϵ to add an extra layer of stochasticity (eq 26):

z=μ+σ⊙ϵ 26

where ϵ ∼ *N*(0, *I*), and *I* is the identity matrix of a Gaussian distribution.

Now that the variational posterior and sampling process are defined, the latent vector *z* can be sampled and sent through the decoder to reconstruct the input *x̂*. The loss function (eq 27) for training both models is a combination of the reconstruction loss, which measures the decoder’s ability to reconstruct the sample, plus the Kullbach–Leibler (KL) divergence.^115^ The KL divergence is a metric applied to measure the distance between two distributions. This metric is always non-negative and is minimized during training. Minimizing the KL divergence is equivalent to minimizing the negative log-likelihood, which is equivalent to maximizing the log-likelihood. It is common in machine learning to express metrics in a form where the goal is to minimize it. Combining a reconstruction loss metric for continuous data such as MSE, with the KL divergence to measure the distance between the learned posterior distribution and the assumed prior, gives us the following loss function:

$$L=\frac{1}{N}\overset{N}{\underset{i=1}{\sum }}{\left(x_{i}-\mathrm{x̂}\right)}^{2}+D_{\mathrm{KL}}\left(q_{\theta }\left(\mu ,\sigma \right)||N\left(0,1\right)\right)$$ 27

where *x*_*i*_ is the real input, *x̂* is the composite output of *f*(*g*(*x*_*i*_)), and *D*_KL_(.) is the KL divergence added to regularize the output by shifting the posterior distribution toward the assumed prior, conditioning the latent space to follow a Gaussian distribution *N*(0, 1).

In summary, training a VAE consists of the following steps: (i) passing observed data *X* through the encoder function *g*(.) to compute the mean μ and standard deviation σ of the approximate posterior distribution (latent space), (ii) applying the reparametrization trick as described in eq 26, (iii) feeding the latent vector *z* through the decoder function *f*(.) to perform signal reconstruction, (iv) computing the total loss as the sum of the reconstruction loss and KL divergence loss, and (v) backpropagating the error to optimize the network parameters. After training is finalized, the encoder is no longer required for generating new samples. New synthetic data can be produced by sampling latent vectors from the assumed prior Gaussian distribution *N*(0, *I*) and passing them through the decoder. To demonstrate this process, examples of training a VAE are available in the complementary code repository (see the Data and Software Availability section below). The examples highlight various approaches to handling the data preprocessing, changing the training paradigm and loss functions to account for specific requirements, and illustrating regularization procedures such as KL annealing and gradient clipping to prevent posterior collapse and improve training stability.^116^

The strength of the VAE is embedded in its simple design and training methodology and its ability to model continuous latent spaces. Methods originally proposed for image recognition and similar tasks are easily reformulated to work on 1D data, such as spectral signals. This can be achieved via fully connected dense layers that intrinsically work on 1D inputs of continuous data or by translating 2D convolutional operations to their 1D counterparts. Example applications that have been applied in spectroscopy include sparse autoencoders for dimensionality reduction and feature learning,^117,118^ denoising autoencoders (DAE) for noise reduction and generating smoother data,^119^ and variational autoencoders (VAEs) for generating synthetic data.^106,107^

### 5.2 Generative Adversarial Networks

Generative adversarial networks (GANs)^17^ are a popular framework for training two models in an adversarial-style two-player game. A generative model *G* is employed to learn a data distribution and generate new synthetic data through a series of mapping transformations from pure noise. A discriminator model *D* is trained to estimate the probability that a sample is real or synthetic, as outlined in Figure 17. The generator *G* aims to produce realistic data capable of tricking *D* into making incorrect predictions, while *D* aims to provide confident predictions when discriminating between them. Once the models reach an equilibrium, where *G* can produce high-quality outputs, and *D*, optimally, is unable to distinguish between the real and fake samples, *D* can be discarded and *G* employed for data generation.

**Figure 17:**
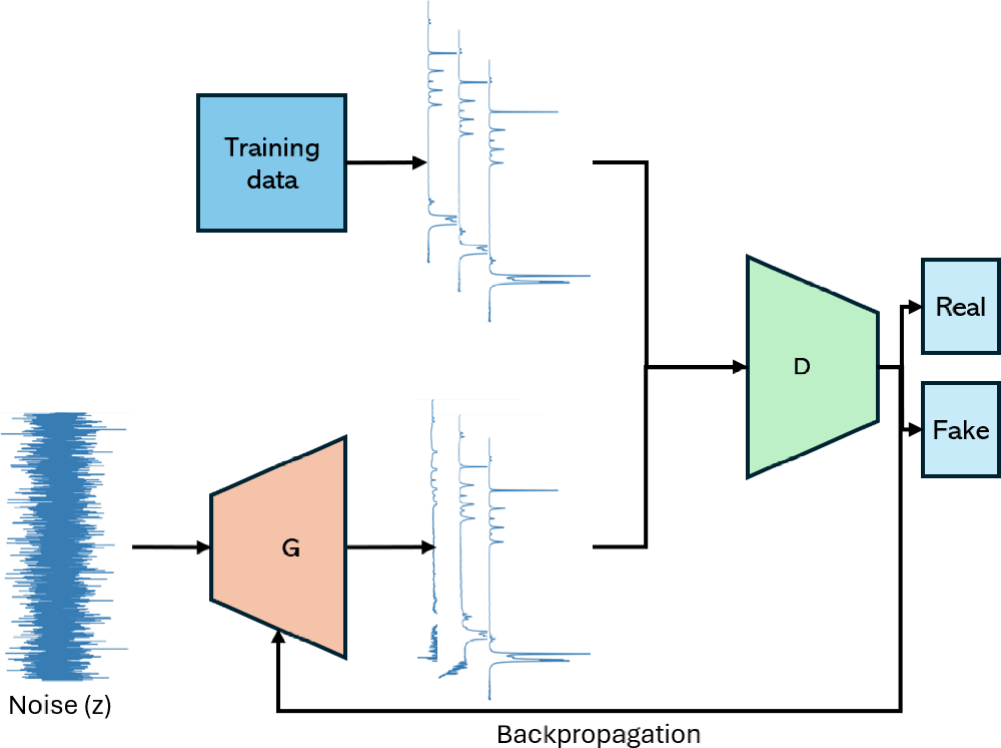
High-level example of GAN architecture.

The training procedure for *G* is optimized to learn a transformation when sampling from a pure noise distribution to a distribution *p*_*g*_(.) over observed data *x*. Analogous to the AE, a simple prior distribution *p*_*z*_(*z*) over latent variables is assumed such as a standard Gaussian. The function *G*(*z*;θ_*g*_) represents the differentiable mapping function from the prior to the generator’s distribution *p*_*g*_(*x*). This function is typically modeled as an FCNN or CNN. During training, the transformation model *G* works to minimize the log-probability that *D* correctly classifies the instance (eq 28):

$$\underset{G}{\min}=E_{z∼p_{z}\left(z\right)}\left[\log\left(1-D\left(G\left(z\right)\right)\right)\right]$$ 28

where *G*(*z*) is the new sample resulting from the transformation of the prior latent variables, *D*(*G*(*Z*)) is the probability output that the instance is real or synthetic, and $E_{z∼p_{z}\left(z\right)}$ represents the expected average value over time.

The probability of an instance being real or synthetic is defined as a differentiable function with a scalar output, *D*(*x*;θ_*d*_), which is also represented as a neural network. The prediction model *D* is trained to maximize eq 28, which simultaneously maximizes its own objective function (eq 29):

$$\underset{D}{\max}=E_{x∼p_{\mathrm{data}}\left(x\right)}[\log\left(D\left(x\right)\right]$$ 29

Here, the model *D* aims to maximize the average expected value of the log-probability that a given sample is real or synthetic. By combining these terms, where *G* attempts to minimize the right-hand side, and *D* attempts to maximize the full objective function, the models are trained simultaneously in an adversarial fashion (eq 30):

$$\underset{G}{\min}\underset{D}{\max}E_{x∼p_{\mathrm{data}}\left(x\right)}\left[\log\left(D\left(x\right)\right)\right]+E_{z∼p_{z}\left(z\right)}\left[1-\log\left(D\left(G\left(z\right)\right)\right)\right]$$ 30

In practice, each iteration in the training process consists of a sampling, transformation, and prediction step. First, the prior distribution is sampled according to the user-defined batch size and dimensions of the latent variables. Next, the latent vectors are sent through *G* to perform a set of transformations and generate new synthetic data. The synthetic data, along with a batch of real data with the same dimensions, are sent through *D* for a prediction to be made on the validity of the new data. Lastly, the errors are calculated on the predictions and the gradients are backpropagated through both models. After training, new samples can be generated following the same routine, by sampling from the prior distribution and passing the data through the generator. We provide working examples of both fully dense and convolutional based GANs in our complementary code repository.

GANs have been found to produce higher quality data in comparison to the VAE, however, this also comes with some caveats. While VAEs are much easier to train, their output quality is not as high. In contrast, GANs can generate high-quality data but are notoriously difficult to train due to issues such as (i) convergence failure due to hyperparameter sensitivity, (ii) mode collapse resulting from the generator missing vital information and patterns, and outputting limited sets of examples, (iii) when the discriminator performs too well it results in diminishing gradients backpropagated to the generator and lower quality generated data, or (iv) increased training time and computational resources required. For these reasons, many extensions have been proposed to optimize the architecture, the objective functions, and the evaluation metrics to try and simplify the training.^120^ In the domain of spectroscopy, GANs have been applied quite successfully to a number of tasks for Raman,^13,121,122^ NIR,^123–125^ ultrafast IR,^126^ and LIBS.^15^ Similar to VAEs, GANs work out-of-the-box for 1D spectral data. If implementing them as CNNs, translation from 2D to 1D layers is very simple and does not require changes to the underlying mathematics, with the exception of some evaluation metrics specifically designed for images.

### 5.3 Normalizing Flows

Normalizing flows (NF)^127^ are flow-based generative modeling techniques designed to learn complex distributions by transforming a simple distribution through a series of differentiable and invertible transformations. The goal is to learn a series of function transformations *f* that map a simple base distribution *p*(*z*), such as a standard Gaussian where *N*(0, *I*), to a complex distribution *p*_θ_(*x*) resembling the observed data. Once the functions are learned, a sample can be drawn from the base distribution and then passed through the sequence of transformations. This process generates synthetic samples from a learned distribution that approximates the true data distribution. In this work, we describe the theory of NFs with a fixed number of transformations, referred to as finite flows (eq 31):

$$x=f_{\theta }\left(z\right)=f_{K}◦\;f_{K-1}◦\;\cdot \cdot \cdot \;◦\;f_{1}\left(z\right)$$ 31

where each *f*_*k*_ is an invertible transformation applied to the previous transformations output, starting at *f*_1_ on a latent variable *z* drawn from a simple base distribution.

The first important concept in this framework is the change-of-variable (CoV) rule. The CoV rule is a technique applied in calculus to simplify an integral or differential equation by substituting existing variables with new ones.^128^ This is achieved by defining a new variable and its differentials, substituting them in place of an existing variable, and solving the equation in its simplified and more tractable form. With regard to NFs, the CoV rule enables the expression of the probability density of a complex distribution *p*_θ_(*x*) as the result of a transformation applied to a simple base distribution *p*(*z*) with a known density.

To apply the CoV rule, a differentiable method to quantify how the probability density changes during the transformation process is required. This leads us to the next important concept; the Jacobian matrix and its determinant.^129^ Recall that when training neural networks backpropagation is performed following the chain rule of calculus in order to compute the gradients of the cost function *J* with respect to the model parameters. For a scalar function *f*(*x*), i.e., a neural network with a single output, this produces a gradient vector ∇*f*, which is a row vector of size 1 × *n* containing the partial derivatives for the input *x* of size *n*:

$$\nabla f=\left[\frac{\partial f\left(x\right)}{\partial x_{1}},\cdot \cdot \cdot ,\frac{\partial f\left(x\right)}{\partial x_{n}}\right]$$ 32

For multivariate vector-valued functions, such as deep neural networks with multiple target outcomes, this defines a function that maps *n* real inputs to *m* real outputs $f:R^{n}\to R^{m}$ . The Jacobian matrix of *f* is an *m* × *n* matrix, where each row represents the partial derivatives of a single output variable *y*_*i*_ with respect to all *n* input variables of *x*, and the columns represent the partial derivatives of all *m* output variables of *y* with respect to a single input variable of *x*. Thus, the Jacobian matrix *J* represents how changes to the input variables of *x* affect the output variables of *y*:

$$J=\left[\begin{array}{llll} \frac{\partial y_{1}}{\partial x_{1}} & \frac{\partial y_{1}}{\partial x_{2}} & \cdots & \frac{\partial y_{1}}{\partial x_{n}}\\ \vdots & \vdots & \ddots & \vdots \\ \frac{\partial y_{m}}{\partial x_{1}} & \frac{\partial y_{m}}{\partial x_{2}} & \cdots & \frac{\partial y_{m}}{\partial x_{n}} \end{array}\right]$$

With regard to the NF framework, the rule for the transformation of densities is defined as a smooth, invertible mapping $f:R^{n}\to R^{n}$ , achieved through applying the CoV rule. This mapping results in an *n* × *n* Jacobian square matrix, and a special property referred to as the determinant, which can be computed when operating on square matrices. The determinant of a Jacobian matrix det­(*J*) is a scalar that quantifies how the volume of the distribution has been scaled. Positive values indicate that the orientation of the space is preserved, while negative values indicate that it was reversed. Instead, the absolute value of the determinant |det­(*J*)| is computed, which characterizes the degree to which the volume of the distribution has expanded or contracted, while ensuring that the result of the transformation remains positive.

Now, according to the inverse function theorem (IFT), if *f* is continuous, differentiable, and its Jacobian determinant at a given point is nonzero, then *f* has a differentiable inverse function *f* ^–1^, such that if *x* = *f*(*z*), then *z* = *f* ^–1^(*x*). The absolute value of the Jacobian determinant of the transformation represents how the probability density was adjusted and scaled by the transformation. By computing this value and applying it in the CoV formula (eq 33), the probability density at each step can measured and adjusted:

$$p_{x}\left(x\right)=p_{z}\left(f^{-1}\left(x\right)\right)|\det\left(\frac{\partial f^{-1}\left(x\right)}{\partial x}\right)|=p_{z}\left(z\right)\left|\det\left(\frac{\partial z}{\partial x}\right)\right|$$ 33

where *z* is a random variable with probability density *p*_*z*_(*z*), $\frac{\partial z}{\partial x}$ is the Jacobian matrix of the inverse transformation *f* ^–1^(*x*), and |det(.)| is the absolute value of the Jacobian determinant. Lastly, the absolute value of the determinant also ensures that the new density is normalized and valid, meaning it integrates to 1 over the new space, hence the name normalizing flow.

The next step is to define the theory with regard to the model training and variational inference. As previously mentioned, variational inference is a method for approximating intractable distributions by modeling it as an optimization problem over an assumed prior. While NFs are not a variational inference method, they can be integrated to improve flexibility. In comparison to VAEs that optimize a lower-bound of the log-likelihood, NFs can calculate an exact log-likelihood of the data. The function *f* is designed to operate over a sequence of transformations, so even if the probability density at any point is complex or unknown, it can naturally be traced back to the initial base distribution. This is achieved by substituting the determinant in the CoV formula (eq 33) with the sum of the log-determinants of the Jacobians for each intermediate step in the sequence, which ensures an exact calculation of the overall log-likelihood. Refactoring eq 33 to include model parameters θ, training is simplified to optimizing the negative log-likelihood (eq 34) on observed data *X*:

$$\log p_{x}\left(x|\theta \right)=\log p_{z}\left(z\right)+\overset{K}{\underset{k=1}{\sum }}\log\left|\det\frac{\partial f_{k}\left(z_{k-1}|\theta \right)}{\partial z_{k-1}}\right|$$ 34

where *x* is the observed data, *p*_*x*_(*x*|θ) is the probability density of *x* learned by the model parametrized by θ, log *p*_*z*_(*z*) is the log-density of the starting base distribution, and the right-hand side is the sum of the log-determinants of the Jacobian matrix for each *z*_*k*_, where *z*_*k*_ = *f*_*k*_(*z*_*k*–1_), that represents the changes introduced locally by the transformation *f*_*k*_.

With regard to the domain of spectroscopy, NF applications have been rarely explored and tend toward astronomy related tasks. Examples include one preprint^130^ and one proceedings^131^ article on density estimation of LIBS data from the Mars rover Curiosity, red-shift retrieval, modeling, and inference,^132–134^ and retrieving exoplanet atmosphere spectra.^135,136^ Other examples include the quality enhancement of super-resolution magnetic resonance spectroscopic imaging for glioma patients,^137^ a few-shot change detection method for 3D HSI data,^138^ and NF extensions GLOW and Neural Spline Flows for modeling stellar spectra.^139^

### 5.4 Diffusion

Diffusion models are a class of score-based generative latent variable models capable of producing high-quality and high-fidelity synthetic data.^18^ Trained via variational inference, diffusion models are defined as parametrized Markov chains designed to progressively add noise to a sample over a selected number of time steps and transform a complex distribution into a simple distribution. A reverse process is learned to remove the noise relative to the time step. In this work, we discuss the most popular enhancement that gained widespread adoption and implementation, the Denoising Diffusion Probabilistic Model (DDPM).^22^

Briefly, Markov chains are stochastic processes for modeling a sequence of events while holding the Markov property. The Markov property states that given a sequence of events, the next event is only dependent on the current state. In the forward diffusion process (eq 35), Gaussian noise is gradually added to the data over *t* successive steps for a total of *T* time steps. The posterior is then a simple distribution constructed as a fixed Markov chain of length *T*, which can be computed as the product of Gaussian noise additions over successive steps *t*:

$$q\left(x_{1:T}|x_{0}\right)\text{:}=\overset{T}{\underset{t=1}{\prod }}q\left(x_{t}|x_{t-1}\right)$$ 35

where *x*_1_, ..., *x*_*T*_ are latent variables with the same dimensions as *x*_0_. At each step *t*, a Gaussian distribution is sampled, conditioned using the mean of the previous distribution at time step *t* – 1 and a fixed variance schedule β_*i*_, ..., and β_*T*_. The variance schedule controls the amount of noise added at each step, allowing for inference on the amount of noise added at any given time step by following the chain. Incorporating the conditional Gaussian noise sampling step (eq 36), the above equation can be then expressed as

$$q\left(x_{1:T}|x_{0}\right)\text{:}=\overset{T}{\underset{t=1}{\prod }}N\left(x_{t};\sqrt{1-\beta_{t}}x_{t-1},\beta_{t}I\right)$$ 36

where $\sqrt{1-\beta_{t}}x_{t-1}$ and β_*t*_ *I* are the mean and variance identity matrix, respectively, of the previous conditional Gaussian in the chain.

The forward process provides a further benefit that it can be represented in closed-form, meaning that the problem can be represented as a finite number of operations with standard mathematical operators. Typically, when computing loss metrics, we apply the Monte Carlo method to calculate the expected values (averages) by repeatedly and randomly sampling. By representing the forward process as α_*t*_ := 1 – β_*t*_ and α̅ _*t*_ := ∏_*s* = 1_^*t*^α_*s*_, i.e., the cumulative product of added noise, the forward process can be computed more efficiently, such that for any step *t*, a noisy sample *x*_*t*_ can be generated:

$$q\left(x_{t}|x_{0}\right)\text{:}=N\left(x_{t};\sqrt{{\mathrm{\alpha ̅}}_{t}}x_{0},\left(1-{\mathrm{\alpha ̅}}_{t}\right)I\right)$$ 37

where *x*_*t*_ is calculated as the combination of *x*_0_ and the added Gaussian noise through successive time steps *t*. An example of the diffusion process is illustrated in Figure 18, demonstrating the successive addition of Gaussian noise.

**Figure 18:**
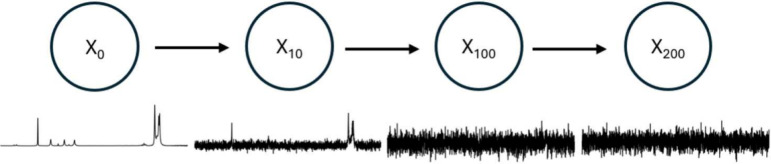
Diffusion process at time steps [0, 10, 100 and 200] applied to Raman spectra of pure cyclohexane (Figure 3).

Now referring to the reverse process, it is defined as a joint distribution *p*_θ_(*x*_0:*T*_) over the latent variables, constructed as a parametrized Markov chain with learned Gaussian transitions. Starting from a pure noise distribution *p*(*x*_*T*_) = *N*(*x*_*T*_;0, *I*), the joint distribution is calculated as the product of conditionals at each step in reverse times the probability density of the starting sample from the pure noise distribution:

$$p_{\theta }\left(x_{0:T}\right)\text{:}=p\left(x_{T}\right)\overset{T}{\underset{t=1}{\prod }}p_{\theta }\left(x_{t-1}|x_{T}\right)$$ 38

A deep learning model attempts to learn the conditional parameters of the Gaussian transitions and gradually remove the noise step-by-step to produce a high-quality sample:

$$p_{\theta }\left(x_{t-1}|x_{t}\right)\text{:}=N\left(x_{t-1};\mu_{\theta }\left(x_{t},t\right),\Sigma_{\theta }\left(x_{t},t\right)\right)$$ 39

where μ_θ_ and Σ_θ_ are the learned mean and variance. The model parameters θ are optimized via variational inference by maximizing the ELBO, similar to the VAE. By training the model to maximize the ELBO, it indirectly minimizes the negative log-likelihood. This is achieved by incorporating and minimizing the KL divergence in the loss function, which as previously mentioned, is equivalent to maximizing the log-likelihood and ELBO. The model thus learns the parameters to reverse the learning process and predict the amount of noise in the data at each step through the following loss function (eq 40):

$$E_{q}\left[D_{\mathrm{KL}}\left(q\left(x_{T}|x_{0}\right)∥p\left(x_{t}\right)\right)+\underset{t>1}{\sum }D_{\mathrm{KL}}\left(q\left(x_{t-1}|x_{t},x_{0}\right)∥p_{\theta }\left(x_{t-1}|x_{t}\right)\right)-\log p_{\theta }\left(x_{0}|x_{1}\right)\right]$$ 40

where $E_{q}$ is the long-term average expected value of the KL divergence *D*_KL_(*q*||*p*) between the true forward and learned reverse noise posterior distributions, conditioned on *x*_0_ to make it tractable as per eq 37, and −log *p*_θ_(.) is the negative log-likelihood of the first noise step given its next direct step.

The divergences in the loss function are all comparing Gaussians; therefore, it can incorporate Rao-Blackwellization, which is a statistical technique to reduce the variance between estimators by conditioning on subsets of the variables. Instead of calculating estimations for all the latent variables, it is conditioned on the latent variable at each step, and *x*_0_. Such that the true posterior is now:

$$q\left(x_{t-1}|x_{t},x_{0}\right)=N\left(x_{t-1};{ũ}_{t}\left(x_{t},x_{0}\right),{\mathrm{\beta ̃}}_{t}I\right)$$ 41

where

$${ũ}_{t}(x_{t},x_{0})\text{:}=\frac{\sqrt{{\mathrm{\alpha ̅}}_{t-1}}\beta_{t}}{1-{\mathrm{\alpha ̅}}_{t}}x_{0}+\frac{\sqrt{\alpha_{t}}(1-{\mathrm{\alpha ̅}}_{t-1})}{1-{\mathrm{\alpha ̅}}_{t}}x_{t}$$ 42

$${\mathrm{\beta ̃}}_{t}\text{:}=\frac{1-{\mathrm{\alpha ̅}}_{t-1}}{1-{\mathrm{\alpha ̅}}_{t}}\beta_{t}$$

Now that the true posterior for the forward process and the reverse process for training is defined, it can be simplified and implemented for training. The first step is to sample a real spectral signal, and sample a time step uniformly, e.g., *t* = [1, ..., *T*], or define a learning process for the variance schedule. Next, Gaussian noise is sampled where ϵ ∼ *N*(0, *I*) and the forward process is performed to generate the noisy signal *x*_*t*_. The model ϵ_θ_(*x*_*t*_, *t*) is realized as a fully convolutional U-Net architecture with added time embeddings to reconcile the amount of Gaussian noise in the input with the respective time step. The model attempts to quantify the amount of the noise in the input through comparison of the true noise and predicted noise. Using the reparameterization trick from eq 37, the true noise sample can be calculated as

$$x_{t}=\sqrt{{\mathrm{\alpha ̅}}_{t}}x_{0}+\sqrt{1-{\mathrm{\alpha ̅}}_{t}}\epsilon$$ 43

where *t* is the sampled time step, ϵ ∼ *N*(0, *I*), and *x*_0_ is the real sample as input to the model. The training is then reduced to performing a gradient descent over the following loss function:

$$L_{\mathrm{simple}}\left(\theta \right)=E_{t,x_{0},\epsilon }\left[∥\epsilon -\epsilon_{\theta }\left(x_{t},t,x_{0}\right){∥}^{2}\right]$$ 44

where the expected value conditioned on step *t*, real input *x*_0_, and noise ϵ ∼ *N*(0, *I*), is the squared difference between the true noise ϵ and the models prediction, conditioned on noisy image *x*_*t*_ and real input *x*_0_.

Diffusion model frameworks have demonstrated excellent performance in generating very high-quality synthetic images; examples include Stable Diffusion, Imagen, GLIDE, and DALLE-3. The key difficulty with diffusion models is the implementation and understanding of the underlying theory. The original authors have made their code publicly available for image-based generation; however, refactorization is required to convert the code to work on 1D signals, as opposed to multichannel 2D images. Currently, the research domain for diffusion applications of synthetic data for spectroscopy is sparse and overlaps with literature referencing the physical diffusion phenomena. The most popular research area is tending toward super resolution and image restoration for hyperspectral imaging.^140–145^ Articles utilizing diffusion for spectral data are also available for tasks such as rapid identification of 2D materials using Raman spectra,^146^ snapshot compressive imaging reconstruction for multispectral imaging,^147^ estimating probability densities of galaxy star spectra,^148^ and some nonpeer reviewed preliminary works for discovery of 3D structures of disordered materials,^149^ and predicting optical galaxy spectra from photometric broad-band images.^150^ As a final note, the majority of the references have made their code publicly available; however, they operate over 2D image structures.

### 5.5 Managing Training and Data Integrity

Two important conditions to evaluate during training include the model performance and the quality of the synthetic data. Training generative models can be a resource-intensive and time-consuming task. It is recommended to continually monitor and frequently run validation tests on the model’s learning progress and the synthetic data being produced. In this section, we expand on both of these concepts and describe the options that are available.

#### 5.5.1 Monitoring Training

Monitoring the training progress of the generative models is essential to ensure that they produce meaningful and realistic outputs. The model’s learning performance is directly related to the quality of the synthetic data generated. Regular evaluation allows researchers to detect issues early in the training process and prevent unnecessary resource consumption. For example, a common challenge in deep learning is overfitting, which leads to poor performance and poorly generated data. For VAEs and GANs, this presents as mode and posterior collapse, respectively, where repeating and nonrepresentative samples are produced. In the Diffusion and NF transformations, overfitting manifests as memorization of the training data, resulting in poor generalization and low sample diversity.

By monitoring metrics such as the loss function, diversity of generated samples, and convergence trends, researchers can fine-tune hyperparameters and adjust the training strategy to prevent overfitting. This process differs depending on the generative method and the type of data. It is important to understand the loss function applied to accurately evaluate the training progress. For example, MSE is employed to measure the reconstruction loss in VAEs, and also to measure the difference between the predicted noise and the true noise in the Diffusion model. However, VAEs also incorporate the KL divergence term. In this case, both values can be logged separately and with their sum, i.e., the full loss, to determine which is contributing most to the overall error. The frequency and modularity of logging the training results can also be adapted. As training progresses, it is recommended to log the loss value every iteration, a few iterations, or epoch. The way the loss is recorded depends on the users preference, difficulty of learning, and size of the data set. For difficult problems, it may be more suitable to log in smaller iterations and track early convergence (overfitting). For large data sets, it will be less computationally intensive to calculate the loss every epoch. In the practical examples provided, the loss for both the generator and discriminator is logged for every batch, since each batch contributes to the model’s learning progress. This allowed for early identification of mode collapse, where the discriminator learned too quickly so the gradient updates were small and the resulting generated data were not meaningful.

An alternative method to evaluating the model’s progress is through producing visual outputs of synthetic data every few epochs. This allows us to visualize the structure of the synthetic data as the model progresses. Coupling this with frequent model checkpointing, which stores the model’s current state and weights to disk, enables researchers to select and load an earlier version of the model after overfitting is identified. In our practical code examples, we demonstrate how noise can be sampled and passed through the VAE decoder and the GAN generator during the training process. Synthetic data are generated and plotted for visual evaluation at each checkpoint. Since the GANs training process is more difficult, this is also paired with storing the model weights and losses to disk. Lastly, early stopping criterion can be applied to automatically stop training. Early stopping is a regularization technique designed to prevent overfitting by monitoring validation metrics, such as loss and accuracy, and terminating training when they begin to degrade. The model’s performance is continuously evaluated on a separate validation set, and training is stopped after a user-defined patience threshold is exceeded, such as when the validation loss increases for five consecutive epochs. This prevents the model from memorizing the training data and overfitting, ensuring it still generalizes well to new data.^151,152^

#### 5.5.2 Validating Synthetic Data

Validating artificially generated data is a crucial step to ensure that they are faithful and representative of real-world measurements. The simplest method is to apply some statistical comparisons between real and synthetic data. For instance, comparing the mean, median, and standard deviations between individual samples, such as those reconstructed by a VAE. Library search procedures for comparing a spectrum directly, or to groups of spectra, in a database has been a vastly explored area of research. These define a class of attribute-based similarity metrics such as correlation coefficients calculated on either mean-centered or direct intensity values and vector-based metrics like the Euclidean and Manhattan distance, and cosine similarity.^153,154^ Other specialized metrics have been proposed that capture finer details in the signal’s structure and can account for issues such as mixtures of substances or arbitrary lengths of the signals being compared. These include the discrete Fréchet distance (dFid), longest common sub-sequence^153^ and spectral linear kernel^155^ for example. It is recommended to select and validate a representative spectral matching algorithm on the real data first to ensure that it measures the similarity between samples appropriately.

To complement a quantifiable metric, machine learning models for inference and dimensionality reduction have been applied to compare similarities between synthetic and real data. If a robust model can be developed on only real data, then it can be applied for inference to determine if it can accurately discriminate the synthetic sample as being part of the target class or having a similar concentration. However, this method is less reliable, and as mentioned, it should be applied as a complementary approach to benchmark the selected augmentation or generative methods employed. In contrast, visual-based dimensionality reduction algorithms allow for experts to judge the similarity of synthetic data based on cluster location and density in lower dimensional spaces. Methods such as t-Distributed Stochastic Neighbor (t-SNE) embedding^156^ and Principal Component Analysis (PCA)^157^ have the ability to learn and map high-dimensional data to lower 2D or 3D dimensional latent spaces. t-SNE is a stochastic technique that attempts to create and optimize a mapping of pairwise similarities of the data in high- and low-dimensional spaces. The goal is to preserve local structure and project the data to a lower-dimensional space for easy visualization. In contrast, PCA projects data to lower-dimensional spaces in a deterministic manner, meaning it will provide the same mapping each time it is modeled on the same data. It does this by computing weighted linear combinations of the features, called principal components (PCs), where each component represents a maximization of the data sets variance in a single orthogonal direction. After calculating the PCs, scores for each sample are calculated by projecting the data onto them. Both techniques develop clusters of the data in lower-dimensional spaces such that similar samples should appear clustered together and the clusters should have high density. After augmenting synthetic data, a PCA or t-SNE model that was fit on the original training data can be applied to transform the newly generated data to the lower-dimensional space.^107,158–161^ The new data should ideally be positioned close to their representative target class or concentration-based clusters. Larger spaces between the clusters and samples indicate that the data are less similar and therefore not representative.

The last concern when augmenting synthetic data is justifying the proportion of data introduced during calibration. Increasing training data with synthetic data to achieve a top-level performance is not appropriate if it does not fully represent realistic outcomes. At some point, the newly generated data will neither add new variability nor be representative of the real data. After applying some appropriate metrics to judge the quality of the data, the most representative samples can be augmented, and the calibration model’s learning trends can be observed. Quantification of how much augmented data is required ultimately depends on the quality and amount of real data and the specific problem being evaluated. However, the appropriate amount of synthetic data can be determined by observing the learning trends of the inference algorithms employed by slowly increasing the batch sizes of augmented synthetic data and identifying when the models converge.^107^ Once the models converge, an upper bound on the quantity of synthetic data required can be identified. At this point, adding more data will not improve performance, and it will most likely contribute to overfitting and poorer generalization to new data. In the event that augmentation or generative AI is not feasible and the resulting models still perform poorly, or if strict conditions are required to avoid unrealistic artifacts, physics-based and computational simulation approaches should be considered. Many methods have been proposed to simulate the interactions between electromagnetic radiation and matter, which are grounded in physical laws. Examples include Density Functional Theory (DFT) for quantum mechanical simulations,^162,163^ the Transition Matrix Method (T-Matrix) for wave scattering of complex-shaped particles,^164^ the Transfer Matrix Method (TMM) for wave propagation in layered optical structures,^165,166^ Finite-Difference Time-Domain (FDTD) for solving Maxwell’s equations,^167,168^ Ray-Tracing for geometric optics modeling,^169^ and Discrete Dipole Approximation (DDA).^170,171^

## 6 Conclusion

In this review, we provide a starting point for new researchers in the domains of spectroscopy and generative artificial intelligence. With simplified mathematical explanations and visually intuitive examples of the methods in practice, we endeavored to simplify the process for researchers moving across domains or beginning in both simultaneously. The first key takeaway we wish to highlight is understanding the most common pretreatment and preprocessing methods, drawing attention to their implications and how they relate to deep learning paradigms. Preprocessing methods have a large influence on the performance of both traditional chemometrics and deep learning models and should typically be evaluated for every problem undertaken. The second key takeaway is to consider applying data augmentation methods as opposed to their generative counterparts. While these methods may not perform to the same standard, they still provide a lot of benefits, such as the time and resources required for implementation and higher interpretability of the resulting data. Furthermore, low sample data sets may benefit more from statistical augmentations as opposed to data approximation due to the lack of variation in the underlying distribution. Lastly, effort should be invested from the beginning of the training process to ensure that the data produced is both high in quality and representative, pay close attention to the training progress, and run frequent validation tests. This will prevent excessive resource consumption and allow observations to be made that can improve training efficiency and results.

In regard to the future directions of the field, the main point emphasized from the literature is the lack of research available in state-of-the-art generative methods for spectroscopy. The development of generative artificial intelligence applications has steadily moved toward complex posterior approximation methods such as VAEs, NFs, and Diffusion models. The available work regarding GANs is plentiful; however, the well documented instability of GANs outlines that the implementation and model training will most likely outweigh the output quality. We hypothesize that the domain would benefit from further research into generative flow-based and diffusion models. For example, many flow-based frameworks, such as NICE, RealNVP, GLOW, and Neural Spline Flows have not been explored. Similarly, diffusion model applications for generating synthetic data are underwhelming when considering their popularity for image-based tasks. In contrast to GANs and VAEs, these methods can compute exact log-likelihoods and have more stable training procedures, resulting in better approximations of complex distributions, such as high-dimensional spectroscopy data.

## Data Availability

The data and code supporting the graphical illustrations and practical examples mentioned in this article are available on Github.^172^ For the purpose of Open Access, the author has applied a CC BY public copyright license to any Author Accepted Manuscript and Software arising from this submission.
